# *Z*-Guggulsterone Is a Potential Lead Molecule of Dawa-ul-Kurkum against Hepatocellular Carcinoma

**DOI:** 10.3390/molecules27165104

**Published:** 2022-08-11

**Authors:** Meenakshi Gupta, Shaikh Maryam Ghufran, Tasneem Kausar, Rafat Ali, Subhrajit Biswas, Shahid M. Nayeem, Romana Ishrat, Sher Ali, Ajaz Ahmad, Irfan A. Rather, Maryam Sarwat

**Affiliations:** 1 Amity Institute of Pharmacy, Amity University, Noida 201313, Uttar Pradesh, India; 2 Amity Institute of Molecular Medicine & Stem Cell Research, Amity University, Noida 201313, Uttar Pradesh, India; 3 Department of Chemistry, Aligarh Muslim University, Aligarh 202001, Uttar Pradesh, India; 4 Centre for Interdisciplinary Research in Basic Sciences, Jamia Millia Islamia, New Delhi 110025, Uttar Pradesh, India; 5 Era University, Sarfaraz Ganj, Hardoi Road, Lucknow 226003, Uttar Pradesh, India; 6 Department of Clinical Pharmacy, College of Pharmacy and King Saud University, P.O. Box 2457, Riyadh 11451, Saudi Arabia; 7 Department of Applied Microbiology and Biotechnology, Yeungnam University, Gyeongsan 38541, Korea

**Keywords:** molecular docking studies, MD simulation, guggulsterone, hepatocellular carcinoma, Dawa-ul-Kurkum, ER stress

## Abstract

An ancient saffron-based polyherbal formulation, Dawa-ul-Kurkum (DuK), has been used to treat liver ailments and other diseases and was recently evaluated for its anticancer potential against hepatocellular carcinoma (HCC) by our research team. To gain further insight into the lead molecule of DuK, we selected ten active constituents belonging to its seven herbal constituents (crocin, crocetin, safranal, jatamansone, isovaleric acid, cinnamaldehyde, coumaric acid, citral, guggulsterone and dehydrocostus lactone). We docked them with 32 prominent proteins that play important roles in the development, progression and suppression of HCC and those involved in endoplasmic reticulum (ER) stress to identify the binding interactions between them. Three reference drugs for HCC (sorafenib, regorafenib, and nivolumab) were also examined for comparison. The in silico studies revealed that, out of the ten compounds, three of them—*viz*., *Z*-guggulsterone, dehydrocostus lactone and crocin—showed good binding efficiency with the HCC and ER stress proteins. Comparison of binding affinity with standard drugs was followed by preliminary in vitro screening of these selected compounds in human liver cancer cell lines. The results provided the basis for selecting *Z*-guggulsterone as the best-acting phytoconstituent amongst the 10 studied. Further validation of the binding efficiency of *Z*-guggulsterone was undertaking using molecular dynamics (MD) simulation studies. The effects of *Z*-guggulsterone on clone formation and cell cycle progression were also assessed. The anti-oxidant potential of *Z*-guggulsterone was analyzed through DPPH and FRAP assays. qRTPCR was utilized to check the results at the in vitro level. These results indicate that *Z*-guggulsterone should be considered as the main constituent of DuK instead of the crocin in saffron, as previously hypothesized.

## 1. Introduction

Of all the primary liver cancers, hepatocellular carcinoma (HCC) is considered the most common (~90%) and severe type worldwide. It is considered the third major cause of cancer mortality and the sixth most common malignancy among humans [1,2]. Progression of HCC is a very complex multi-stage process in which alterations in the critical genes regulate the initiation, progression and suppression of the tumor. These genetic modifications take place slowly and drive the progressive transformation from the pre-malignant stage to the dysplastic stage and finally to the malignant stage [3,4]. Encouragingly, due to the recent advancements in science and functional genomic approaches, scientists have identified the major cancer-driving genes and associated oncogenic signaling pathways involved in HCC. More specifically, these genes linked to cascades (RAF, ERK/MAPK, PI3K/AKT/mTOR, WNT/β-catenin, JAK/STAT, ubiquitin-proteasome and hedgehog signaling) play important roles in the pathogenesis and suppression of HCC [5,6].

The latest research also focuses our attention on the strong link between the highly intertwined UPR pathways of endoplasmic reticulum (ER) stress and tumor pathogenesis in several cancer types, including HCC. The ER is a distinctive, multifunctional intercellular organelle involved in lipid biosynthesis and metabolism, protein folding, secretion of proteins and calcium homeostasis. It also transduces signals by cross-talking with other organelles, such as the Golgi apparatus, mitochondria, lysosomes and nucleus. Protein folding requires reticular chaperones localized in the ER. These chaperones ensure that the newly synthesized proteins are folded correctly and are of optimum quality. However, various intracellular and extracellular stimuli can lead to excessive accumulation (termed ER stress) of unfolded and misfolded proteins in the ER. Under such conditions, the accumulated proteins are sensed by three transmembrane proteins (ATF6, IRE1 and PERK) and the unfolded protein response (UPR) is activated with the aim of reinstating normal ER functioning [7]. Normally, the UPR performs cytoprotective functions, such as cell cycle arrest, regulation of protein synthesis and upregulation of UPR-responsive genes. However, in cases of irredeemable or chronic ER stress, this UPR activates pro-apoptotic signaling pathways [8,9]. ER stress is further triggered by characteristic stimuli of solid tumor microenvironments, such as acidosis, hypoxia, reactive oxygen species and nutritional deficiency, as tumor cells have an excessive, mutation-driven need for protein synthesis and high metabolic rates. 

Therefore, in an era of precision cancer medicine, targeting relevant genetic alterations is important to stratify patients and develop molecularly targeted therapies. 

Various plant polyphenols and extracts have been suggested to improve the quality of life and prolong the survival of cancer patients [10]. Dawa-ul-Kurkum (DuK) has been in use for centuries, as mentioned in multiple ancient texts. The name Dawa-ul-Kurkum” is derived from the Persian language, and the main constituent of this medicine is “kurkum”; i.e., saffron. It is recommended for liver dysfunction, anorexia, ascites and abdominal pain. The metabolites present in the herbal constituents of DuK are crocin, crocetin, safranal (*Crocus sativus*), jatamansone, isovaleric acid (*Nardostachys jatamansi*), cinnamaldehyde, coumaric acid (*Cinnamomum cassia*, *C. zeylanicum*), citral (*Cymbopogon jwarancusa*), guggulsterone (*Commiphora wightii*) and dehydrocostus lactone (*Saussurea lappa*). In search of the lead molecule of Duk, we used a structure-based in silico molecular docking approach with AutoDock Vina and predicted the interactions between the pharmacologically active components of DuK and the 32 selected proteins. Based on a literature search, these proteins were selected in accordance with their roles in HCC development (2), progression (14), suppression (6) and ER stress (10). The results were then validated using in vitro studies, MD simulations and qRTPCR.

## 2. Results

### 2.1. Molecular Docking Studies of the Active Constituents of DuK

To validate the docking conditions and assess the accuracy before virtually screening the DuK constituents, each protein was retrieved from its co-crystallized complex and re-docked using AutoDock Vina software against the relevant target. This was undertaken to ensure that the interactions during re-docking were similar to the interactions produced in native conformations by ligand. Then, a docking score for each active constituent was calculated to estimate its free energy of binding towards these proteins (Supplementary Materials Tables S1 and S2). Based on these values, we shortlisted three DuK constituents (*Z*-guggulsterone, dehydrocostus lactone and crocin), as they performed better than others. Further, when the docking results for the three reference compounds interacting with the above proteins were compared, we found that regorafenib performed better than the others and, therefore, regorafenib was considered for further comparisons (Supplementary Materials Table S3).

**Figure 1:**
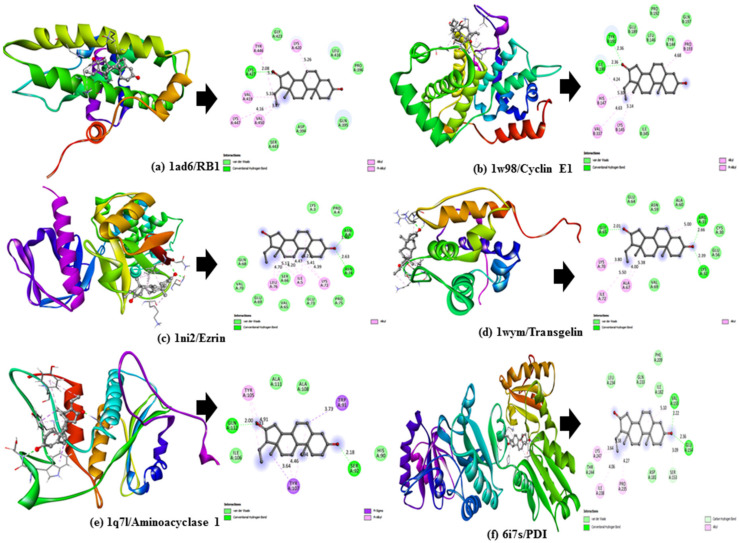
Three-dimensional (3D) docking poses (**left**) and two-dimensional (2D) interaction diagram (**right**) for *Z*-guggulsterone complexed with (**a**) RB1, (**b**) Cyclin E1, (**c**) Ezrin, (**d**) Transgelin, (**e**) Aminoacylase 1, and (**f**) PD1.

The docking score values obtained for the selected DuK compounds (*Z*-guggulsterone, dehydrocostus lactone and crocin) were compared between each other and to the scores of the selected reference compound (regorafenib) for each protein. We observed that *Z*-guggulsterone produced better results than the majority of other DuK constituents, including crocin. Among the HCC proteins, *Z*-guggulsterone showed good interactions with cyclin E1, transgelin, ezrin, RB1 and aminoacylase 1 based on its binding affinity (≥−7.5 Kcal/mol) and, among ER stress proteins, PDI demonstrated better results (Table 1). The binding poses showed that *Z*-guggulsterone forms hydrogen bonds with the key residues of all these proteins. Hydrogen bonding interactions with Ile190 and Tyr 191 of cyclin E1; Arg31, Lys32 and Gly65 of transgelin; Asn6 and Asn74 of ezrin; Lys427 of RB1; Ser92 and Gln112 of aminoacylase 1; and Glu154 and Val155 of PDI were displayed by *Z*-guggulsterone (Figure 1).

As predicted by the docking model, *Z*-guggulsterone showed higher affinity than regorafenib for some proteins (transgelin, S100A14, prohibitin, ezrin, AXIN1, CRT and P58) based on the comparison of their binding affinities. *Z*-Guggulsterone showed a stronger binding score with transgelin (−7.7 kcal/mol) compared to regorafenib (−7.6 kcal/mol). The docking scores with axin 1 indicated that guggulsterone (−8.4 kcal/mol) was superior to the control inhibitor regorafenib (−7.5 kcal/mol). The Ki of guggulsterone for axin 1 was estimated at 0.696 µM (the reference was 3.76 µM). Guggulsterone also showed strong predicted binding with ezrin (−8.7 kcal/mol) compared to the control inhibitor regorafenib (−8.6 kcal/mol). Guggulsterone showed good affinity for prohibitin (−6.5 kcal/mol) and ranked better than regorafenib (−6.4 kcal/mol). Guggulsterone exhibited a stronger binding score (−8.1 kcal/ mol) for S100A14 compared to regorafenib (−8.0 kcal/mol). Moreover, guggulsterone also exhibited strong binding energy with the ER stress protein CRT, with a score (−8.2 kcal/mol) and predicted Ki (0.975 µM) that were much better than the reference compound regorafenib (BA: −7.5 kcal/mol, = Ki: 3.17 µM). Another ER stress component with which guggulsterone demonstrated equal binding energy as regorafenib was P58 (Table 1).

### 2.2. Preliminary Screening of Selected Active Constituents of DuK in Liver Cancer Cells

**Figure 2:**
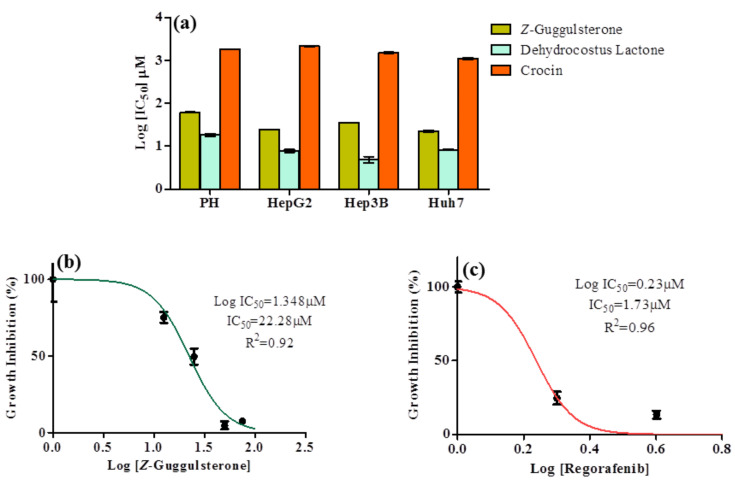
Cytotoxic effect of active constituents of DuK on human hepatocellular carcinoma cell line (Hep3b, Huh7 and HepG2) and normal liver cells (primary hepatocytes). (**a**) Results of cell viability assay when cells were exposed to different concentrations of *Z*-guggulsterone, dehydrocostus lactone and crocin for 24 h. After exposure, IC_50_ values for each treatment were determined based on the spectrometry of formazan formation, and the mean log IC_50_ value was plotted. The y-axis represents the log IC_50_ (in μM) and the x-axis represents the cell lines used. We observed from the comparison that *Z*-guggulsterone is safer than dehydrocostus lactone for parental liver cells. (**b**) Dose–response curves and IC_50_ value for *Z*-guggulsterone in Huh7 cells. They y-axis represents the percentage of normalized absorbance and the x-axis represents the log concentrations of *Z*-guggulsterone. (**c**) Dose–response curves and IC_50_ value for regorafenib in Huh7 cells.

The MTT assay was conducted to evaluate the cytotoxic potential of *Z*-guggulsterone, dehydrocostus lactone and crocin in liver cancer (HepG2, Hep3B and Huh7) and parental liver cells. Under all the treatment conditions, the cell viability of malignant cells was reduced in a concentration-dependent manner. Post-treatment, the IC_50_ values for *Z*-guggulsterone were found to be 24.87, 35.48 and 22.28 μM for HepG2, Hep3B and Huh7 cells, respectively, and for dehydrocostus lactone they were 7.8, 4.97 and 8.311 μM (Figure 2a). However, the growth of primary hepatocytes was least affected by the treatment with *Z*-guggulsterone (compared to dehydrocostus lactone). For crocin, we observed very high IC_50_ values of 2134.1, 1506.7 and 1100.3 μM for HepG2, Hep3B and Huh7 cells, respectively, along with a reduction in the viability of primary hepatocytes. Huh7 cells were more sensitive to *Z*-guggulsterone, as the IC_50_ was the lowest (Figure 2b). We tested regorafenib as a positive control with Huh7 cells (based on our results from in silico studies) and an IC_50_ value of 1.73 μM was noted (Figure 2c).

**Figure 3:**
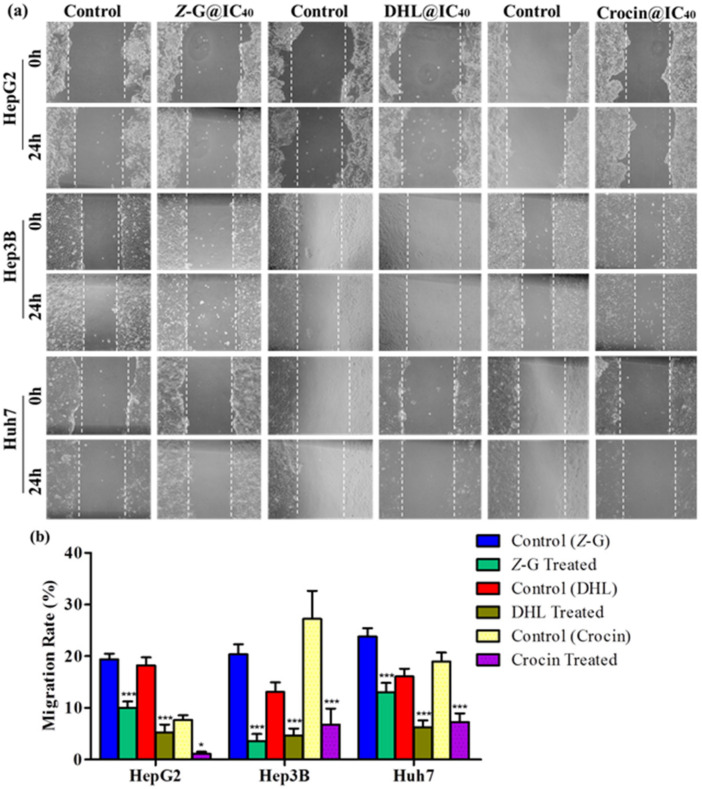
Effects of *Z*-guggulsterone, dehydrocostus lactone and crocin on liver cancer cell migration, assessed by scratch test assay. (**a**) One hundred percent confluent cells were scratched with a pipette tip and treated with the respective IC_40_ value for 24 h. Cells were imaged under a light microscope at 0 and 24 h. (**b**) Cell migration was quantified by measuring wound closure areas at 0 and 24 h. Quantitative data are presented as the means ± standard deviation of three replicates. * *p* < 0.05 vs. its control group; *** *p* < 0.0001 vs. its control group.

We also examined the effects of *Z*-guggulsterone, crocin and dehydrocostus lactone on HCC cell migration through a scratch test assay at the 0 and 24 h time points. The results indicated that these compounds (at their IC_40_ concentrations) significantly reduced the capabilities of cell migration as compared to their respective untreated controls (Figure 3a,b). 

The results of all the above experiments indicated that the active constituent of the plant *Commiphora wightii* (*Z*-guggulsterone) is more potent than the constituents of *Crocus sativus*, contrary to what has been previously hypothesized; hence, we continued further investigation with *Z*-guggulsterone. 

### 2.3. MD Simulation Studies of Z-Guggulsterone with Proteins Shortlisted Based on Molecular Docking

Molecular dynamics (MD) simulations were performed to establish the dynamics of the mechanism used by *Z*-guggulsterone to bind to the proteins under explicit solvent conditions. Here, we performed MD simulations of the docked protein–guggulsterone complex for 50 ns. Based on the results for the binding efficiency, the Ki values, the molecular interactions of *Z*-guggulsterone and its comparison with regorafenib, the following proteins were selected for MD simulation: cyclin E1, ezrin, RB1, aminoacylase, transgelin, S100A14, prohibitin and AXIN1, among the HCC proteins; and PDI, CRT and P58, among the ER stress proteins.

**Figure 4:**
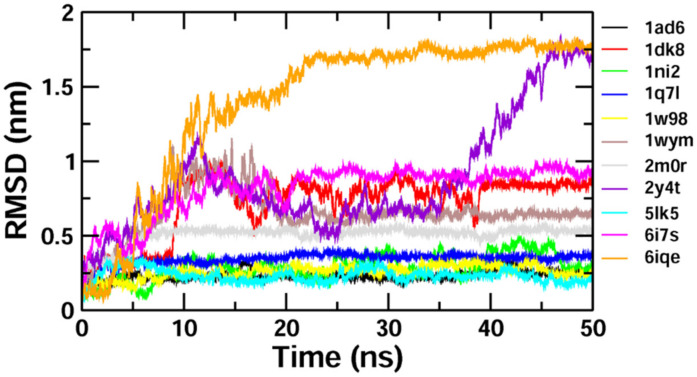
RMSD plots for the protein–ligand complexes over a period of 50 ns. The x-axis represents the MD simulation time (ns) and the y-axis represents the RMSD (nm).

**Figure 5:**
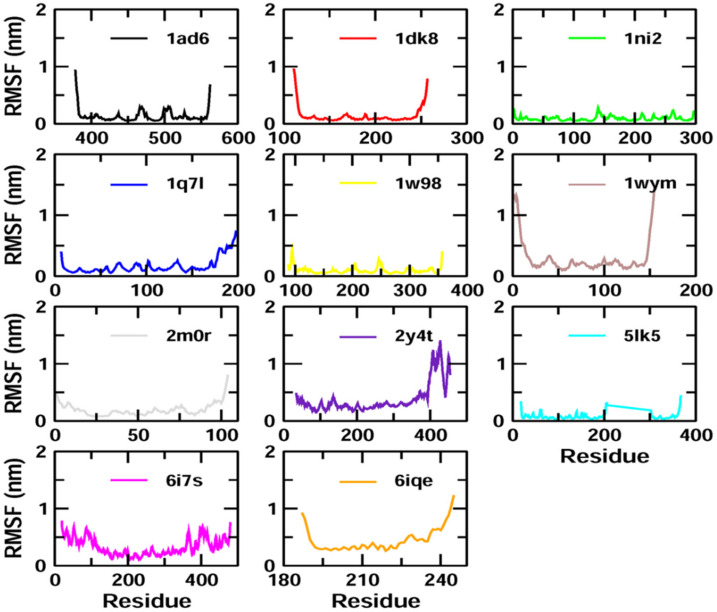
RMSF plots of the protein–ligand complexes with the residue number represented on the x-axis and RMSFs in nm on the y-axis.

**Figure 6:**
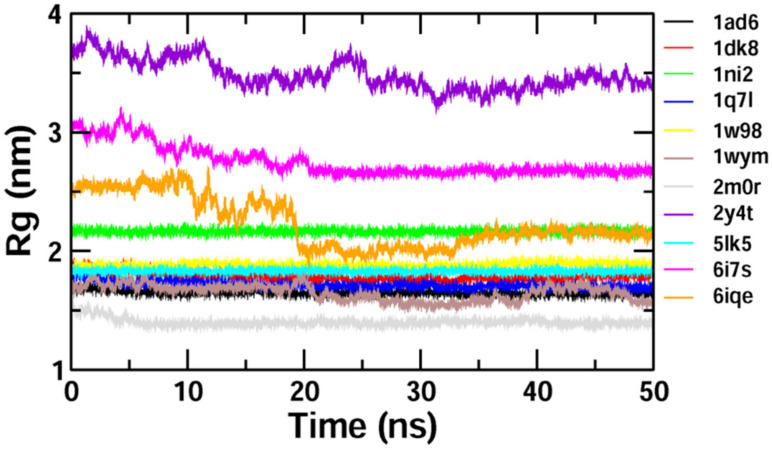
Plot of the radius of gyration (Rg) during the 50 ns molecular dynamics simulation for selected proteins complexed with *Z*-guggulsterone.

The binding stability and conformational changes induced in proteins after binding with *Z*-guggulsterone were evaluated (Supplementary Materials Figure S1). The binding of small molecules in the pocket of a protein brings large conformational changes, and these variations in the structure and stability of proteins were measured using the RMSD, as shown in Figure 4**.** Lower RMSD values (<0.5 nm) for protein–guggulsterone, as in the case of aminoacylase 1 (1q7l), RB1 (1ad6), ezrin (1ni2), cyclin E1 (1w98) and calreticulin (5lk5), suggest the stable binding of the *Z*-guggulsterone at the binding pocket. The average root-mean-square fluctuations (RMSFs) for the *Z*-guggulsterone–protein complexes are plotted in Figure 5 and the radius of gyration (Rg) is shown in Figure 6. 

### 2.4. Free Binding Energy Calculations

To estimate the free binding energy of *Z*-guggulsterone in the docked complex, the entire trajectory acquired during the MD simulations was utilized. For each protein–ligand complex, the van der Waals energy (E_vdw_), electrostatic energy (E_elec_), polar solvation energy (G_polar_), SASA and free binding energy (ΔG_bind_) were calculated. As shown in Table 2, the binding energy of *Z*-guggulsterone in four of the complexes (out of five) was below zero (negative) and, therefore, showed good affinity for the proteins (aminoacyclase 1 (1q7l), RB1 (1ad6), ezrin (1ni2) and calreticulin (5lk5)). Interestingly, van der Waals interactions represented the greatest share of the binding energy in all the complexes studied.

### 2.5. Anti-Oxidant Potential of Z-Guggulsterone through Radical Scavenging Activity and Its Reduction Potential

**Figure 7:**
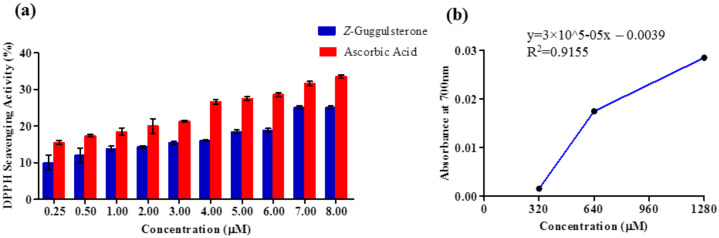
Antioxidant activity of *Z*-guggulsterone as evaluated by (**a**) DPPH and (**b**) FRAP assays.

The DPPH radical scavenging activity of *Z*-guggulsterone was positively correlated with its concentration. At 0.25 μM, its scavenging activity was 10%, and it reached a maximum of 25.09% at 7 μM, after which saturation was attained. For ascorbic acid (used as a positive control), the scavenging activity at 0.25 μM was 15.47%, and it was 33.5% at 8 μM (Figure 7a). Next, the anti-oxidant potential of *Z*-guggulsterone was measured using a FRAP assay. It was observed that the absorbance was positively correlated with the concentration. The absorbance increased from 0.01 to 0.03 as the concentration increased from 320 to 1280 μM (Figure 7b).

### 2.6. Z-Guggulsterone Inhibits Clone-Forming Potential of Liver Cancer Cells and Modulates Cell Cycle Progression

**Figure 8:**
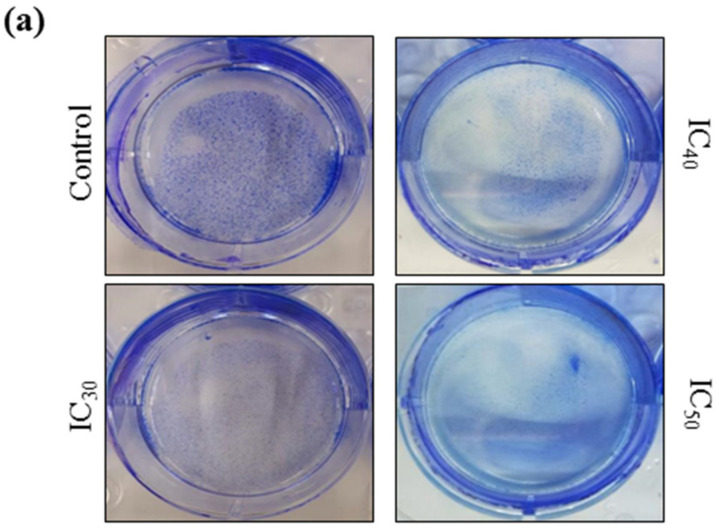

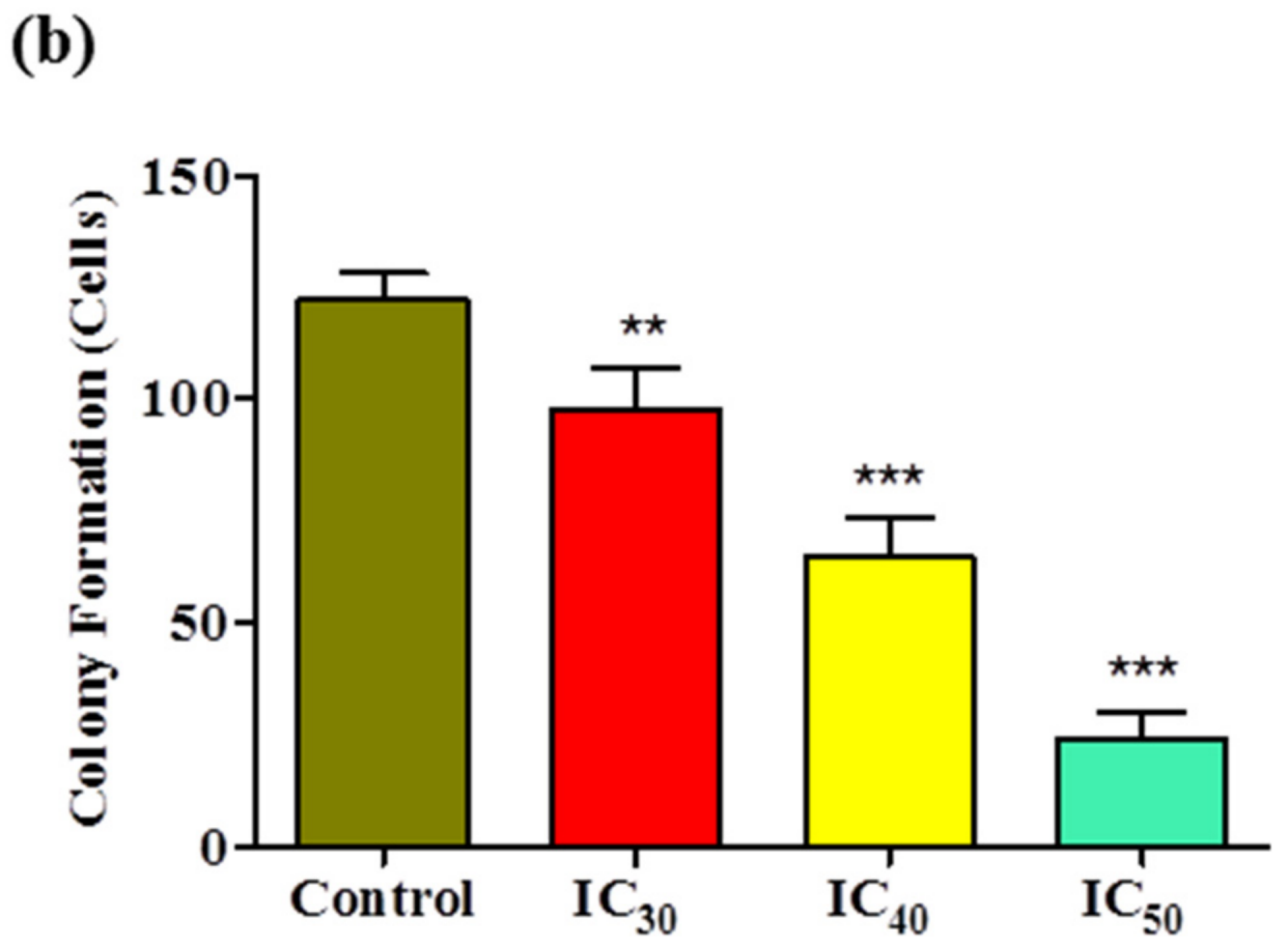
The colony-forming efficiency assay for *Z*-guggulsterone. (**a**) A representative image of the colony formation after staining with 1% Coomassie brilliant blue. (**b**) The numbers of colonies were counted and the results are presented as the means ± standard deviation of three independent experiments. ** *p* < 0.001 and *** *p* < 0.0001 vs. control group.

**Figure 9:**
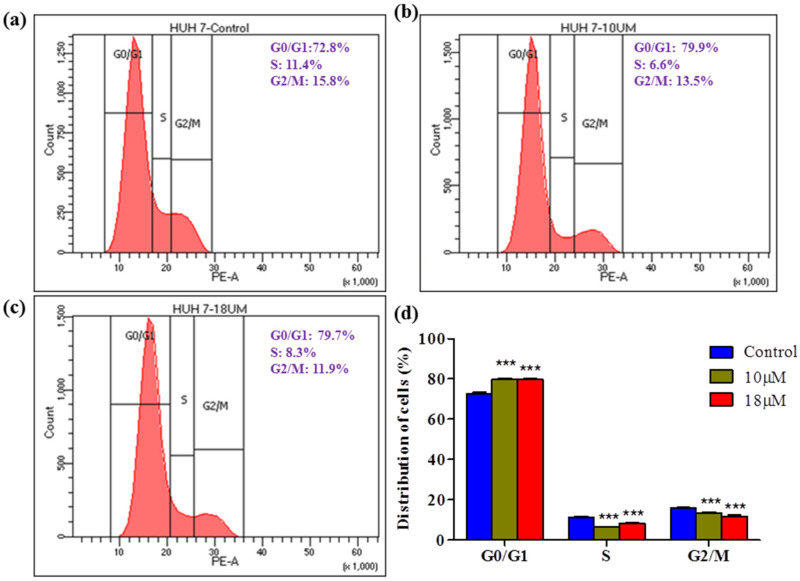
Effect of *Z*-guggulsterone on cell cycle progression. Huh7 cells were treated (**a**) without or (**b**,**c**) with *Z*-guggulsterone (10 and 18 μM). After 24 h of treatment, the distribution of cell cycle phases was quantitated based on flow cytometric analysis. (**d**) Bar diagram showing the percentage of cells present in different phases of the cell cycle. *** *p* < 0.001 vs. its control group.

A colony formation assay was carried out on Huh7 cells to determine the long-term cytotoxicity of *Z*-guggulsterone. The results revealed that *Z*-guggulsterone dose-dependently inhibited the colony-forming potential of Huh7 cells (Figure 8a). We observed that *Z*-guggulsterone at IC_30_ reduced the growth of colonies by more than 20%, while the treatment with *Z*-guggulsterone at IC_40_ decreased the colony formation by more than 47%, in comparison with the control group. When the cells were treated with IC_50_, the population density dropped to 19% of that of control cells (Figure 8b). Our results for the cell cycle analysis showed that *Z*-guggulsterone dose-dependently modulated Huh7 cell cycle progression through a G0/G1 phase increase combined with G2/M depletion (Figure 9). 

### 2.7. Modulation of Expression of Selected Genes by Z-Guggulsterone in HCC Cells

**Figure 10:**
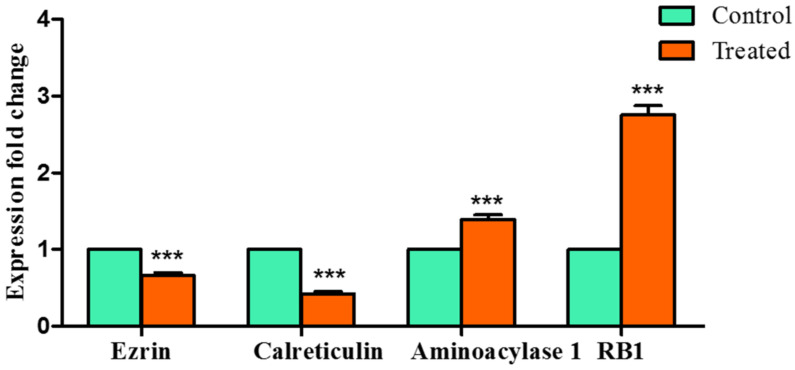
Effects of *Z*-guggulsterone on the expression of genes selected on the basis of MD simulation and free binding energies. *** *p* < 0.001 vs. its control group.

To validate the results obtained from the MD simulation studies, qRTPCR was used to analyze the mRNA levels of these four selected proteins. Huh7 cells were exposed to guggulsterone and the expression was noted. The expression of *aminoacylase 1* (*t* = 11.91; *p* < 0.001) and *RB1* (*t* = 25.81; *p* < 0.0001) was found to be dramatically upregulated, whereas substantial downregulation in the levels of *ezrin* (*t* = 14.74; *p* < 0.0001) and *calreticulin* (*t* = 30.05; *p* < 0.0001) was noted (Figure 10). 

## 3. Discussion

HCC is most frequently known to progress in the background of chronic liver disease triggered by alcohol abuse, hepatitis B/C virus (HBV/HCV) or metabolic syndrome. Due to its synchronic progression, it is commonly multi-nodular when diagnosed [11,12]. Natural preparations (either as single herbs or polyherbal formulations) are gaining enormous attention in modern healthcare. This may be because of their easy accessibility, low cost, safe usage and diverse biological activities [13]. The complex interactions between the various phytoconstituents of polyherbal formulations help in targeting various deregulated pathways of tumor cells and provide a promising multi-dimensional way to manage cancer [13]. Previously, we reported that DuK exhibited excellent antioxidant activity and significantly reduced the viability of liver cancer cell lines (HepG2, Hep3B and Huh7) without affecting the viability of parent liver cells. DuK was phytochemically standardized using HPTLC with respect to marker constituents present in materials from seven individual plants [14]. DuK was further found to be effective against a diethyl nitrosamine (DEN)-induced HCC model in male Wistar rats. In the present study, we utilized an integrated approach involving molecular docking, dynamics simulation and in vitro studies to uncover the lead molecule of DuK, and this is the first study of its kind. 

Molecular docking is a widely used, structure-based drug-design approach that provides information about the binding mode and the affinity between proteins and ligands [15,16,17]. Ranked binding free energies obtained after docking are not always accurate, but they can be utilized for the selection of new molecules, which can then be experimentally validated [18]. Using molecular docking, we first screened for the binding potential of bioactive compounds from DuK with important targets involved in the development, progression and suppression of HCC and ER stress, and three molecules (*Z*-guggulsterone, dehydrocostus lactone and crocin) were shortlisted from the initial screening. 

Next, to identify the lead molecule (out of the three), cytotoxicity and scratch test assays were performed. Incidentally, our study showed similar results as the existing literature [19,20,21,22]. Similar effects were observed in Hep-2, TU212 and H1299 cells, where dehydrocostus lactone significantly inhibited the viability, migration and proliferation of these cells [23,24]. Treatment of pancreatic cancer cells with guggulsterone also showed similar results [25]. 

Effective anti-cancer therapy should kill cancer cells while protecting healthy cells. In our study, we observed that *Z*-guggulsterone affected the viability of cancerous cells without affecting the parental cell population. However, the normal cell population was affected by the other two compounds and, thus, further research was undertaken with *Z*-guggulsterone.

To validate the results of our docking studies and obtain an idea of the interactions of the protein–ligand complexes in motion, we performed MD simulations for selected proteins (obtained from the docking studies) with *Z*-guggulsterone. This simulation step was carried out to investigate protein flexibility and movement and complex stability, which cannot be achieved using molecular docking [26]. Lower RMSD values for the protein–ligand complexes (as in the case of aminoacylase 1, RB1, ezrin and calreticulin) suggested stable binding of *Z*-guggulsterone at the binding pocket of the protein and indicated a minimal change in protein conformation after *Z*-guggulsterone binding. The results were also supported by the RMSF and Rg value plots. RMSF values provide information about the mobility of local structures in protein cavities during MD simulations [27], and we observed low residual fluctuations with several proteins, confirming that there were minimal changes in protein structures upon binding with *Z*-guggulsterone. Rg can be described as the root-mean-square distance from each atom of the system to its center of mass and it is an indicator of protein structure compactness [26,27,28]. This minimal structural deviation noted in our results was responsible for the stable Rg equilibrium throughout the simulation. Further, the molecular mechanics Poisson–Boltzmann surface area (MM-PBSA) method was utilized to calculate the free binding energy of the top five selected protein–ligand complexes. This method indicates the importance of the relative contributions of the van der Waals energy, the electrostatic energy, the polar solvation energy and the solvent-accessible surface area energy to the binding [29,30]. In our study, the binding energy of four complexes was below zero, therefore indicating good binding affinity. Increased levels of reactive oxygen species (ROS) and associated proteins have been identified in almost all types of cancer, where they play an essential role in tumor development and progression. Maintaining the delicate balance between ROS and antioxidant enzymes is, therefore, necessary for cancer management [31,32]. Here, the anti-oxidant potential was determined using DPPH and FRAP assays, and the results confirmed that *Z*-guggulsterone can scavenge the potential damage.

Cancer is mainly characterized by uncontrolled cell proliferation and invasion; therefore, targeting these processes may prove beneficial for chemopreventive agents. Our results for the colony-formation assay showed that the numbers and sizes of colonies derived from *Z*-guggulsterone-treated cells were markedly smaller compared to the vehicle control group. Our results were similar to those observed in pancreatic cancer cells after treatment with guggulsterone [25]. 

Cell cycle arrest at a specific phase is a common mechanism demonstrated by various herbal cytotoxic agents. In this study, we investigated cycle distribution using flow cytometry. The data showed that the proportion of G0/G1 cells increased in a dose-dependent manner, indicating that *Z*-guggulsterone can induce G0/G1 arrest. In contrast, in similar experiments performed by [22], the authors observed that guggulsterone did not induce significant cell cycle arrest in L-02 cells. 

Encouraged by the above results, we further attempted to validate our study using qRTPCR and observed that *Z*-guggulsterone significantly downregulated the expression of *ezrin* and *calreticulin*, whereas it upregulated the expression of *aminoacylase 1* and *RB1*. *Ezrin* is a member of the ezrin–radixin–moesin (ERM) family and is associated with high invasion and poor prognosis in HCC [33,34]. Overexpression of *ezrin* is implicated in the promotion of HCC cell proliferation, epithelial-to-mesenchymal transition (EMT) progression, metastasis and angiogenesis [33]. Its firm interaction and consequent downregulation in the presence of *Z*-guggulsterone may, therefore, be of great importance. Another tumor promoter that was downregulated after treatment with *Z*-guggulsterone was *calreticulin*. This is a multi-functional molecular chaperone localized in the endoplasmic reticulum. It significantly contributes to Ca^2+^ homeostasis, immune responses, transcriptional regulation and protein folding, as well as cellular functions such as cell proliferation, migration and apoptosis, etc. [35]. *Calreticulin* is a potential biomarker and therapeutic target for various cancers, including HCC, and its overexpression is linked with the acceleration of tumor cell migration and invasion [35]. Next, we observed upregulation in two tumor-suppressor genes: *aminoacylase 1* and *RB1*. Knockdown of *aminoacylase 1* in HCC cells is directly linked to an increase in the cell viability, tumor invasiveness the expression of TGF-β1 and ERK1, which proves its tumor-suppressing potential [36]. Another gene, *RB1*, belongs to a family of three proteins that includes RBL1/p107 and RBL2/p130. It is also a tumor-promoter gene and has been found to decrease in several cancers, including hepatocellular carcinoma [37,38]. Our molecule, *Z*-guggulsterone, strongly interacted with both the proteins and upregulated their expression. 

## 4. Material and Methods

### 4.1. Chemicals

The *Z*-guggulsterone used for the study was procured from Natural Remedies Pvt. Ltd. (Bangalore, India). The dehydrocostus lactone and TRI reagent were acquired from Sigma-Aldrich Co. LLC (St. Louis, MO, USA). We purchased cell culture additives/other consumables, such as Dulbecco Modified Eagle Medium (DMEM), DMEM/F-12, fetal bovine serum (FBS), trypsin–EDTA, penicillin-streptomycin (PenStrep) antibiotic solution, epidermal growth factor (EGF), an FITC annexin V/dead cell apoptosis kit, proteinase K, a High Capacity cDNA kit, RNase, ferric chloride, trichloroacetic acid, disodium hydrogen phosphate potassium ferricyanide and nuclease-free water from Thermo Fisher Scientific (Waltham, MA, USA). Phosphoethanolamine was bought from Tokyo Chemical Industry Co., Ltd. (Chennai, Tamil Nadu, India). Type I collagen peptide, bovine serum albumin (BSA) and dimethyl sulfoxide (DMSO cell culture-grade) were purchased from HiMedia Laboratories Pvt. Ltd. (Mumbai, India). Coomassie Blue was obtained from Genetix BioAsia Pvt. Ltd. (Delhi, India). Sodium phosphate monobasic and thiazolyl blue tetrazolium bromide (MTT) were purchased from Sisco Research Laboratories (SRL) Pvt. Ltd. (Pune, India). 2,2-Diphenyl-1-picrylhydrazyl (DPPH) was obtained from Central Drug House Pvt. Ltd. (Delhi, India). SYBR Green master mix from Applied Biosystems (Foster City, CA, USA), and primers from Universal Biotech (New Delhi, India) were used in the present study.

### 4.2. Docking Studies

#### 4.2.1. Ligand Selection 

The ligands selected for this study were ten well-characterized phytochemicals from the seven herbal components of DuK (Supplementary Materials Figure S2). All chemical structures were retrieved from the PubChem compound database (NCBI) (http://www.pubchem.ncbi.nlm.nih.gov (accessed on 29 October 2020). The structures of the standards (sorafenib and regorafenib) were retrieved from their corresponding PubChem entries, and nivolumab was drawn in ChemDraw 7.0.1. 

#### 4.2.2. Protein Preparation

Each ligand structure was retrieved from the database and the geometry was optimized using MM2 energy minimization. Based on the literature, 32 proteins were selected according to their roles in HCC development (2), progression (14), suppression (6) and ER stress (10). The HCC proteins selected for the study have roles in tumor differentiation, cell proliferation, metastasis, apoptosis, migration, invasion and regulation of cell cycle. Additionally, some of them are involved in regulating various signaling cascades in HCC, such as NF-κB signaling, the tyrosine kinase pathway, the RAF/ERK/MAPK pathway, TGF-β signaling, the Wnt/β-catenin pathway and the Ras and JAK/STAT pathway. The ER stress proteins were selected based on their roles in the downstream pathways of PERK, ATF6 and IRE1 signaling and protein folding (Supplementary Materials Tables S1 and S2). The crystal structures of these proteins were retrieved from the RCSB Protein Data Bank (PDB) database (http://www.pdb.org (accessed on 2 November 2020)). Their PDB ids are given in Supplementary Materials Tables S1 and S2. Each protein was used as a rigid structure and all water molecules and hetero-atoms were removed using BIOVIA Discovery Studio Visualizer v.4.5 (Accelrys).

#### 4.2.3. Grid Box Preparation and Docking 

All file conversions required for the docking study were performed using the open-source chemical toolbox Open Babel v.2.3.283. Grid box parameters were set in such a way as to allow for molecular docking using AutoDock Tools v.1.5.6rc384. The grid box centers and dimension were set according to the protein [39]. The molecular docking was carried out with replicates. Molecular docking calculations with each of the proteins were performed for all compounds using AutoDock Vina v.1.1.281. [39]. We further visually inspected all binding poses for a given ligand, and only poses with the lowest root-mean-square deviation (RMSD) values were considered to gain higher docking accuracy of. The Lamarckian genetic algorithm was used during the docking process to explore the best conformational space for each ligand. All other parameters were set as default.

Ki = Exp[(ΔG × 1000)/(R × T)](1)

where ΔG = docking energy; R = 1.98719 cal K^−1^ mol^−1^; and T = 298.15 K.

The lowest binding free energy (i.e., the best score for the docking pose with the lowest RMSD) was used to predict the highest ligand/protein affinity. The AutoDock Vina docking scores of these selected constituents (which ranked higher among the phytoconstituents or better than the control inhibitor) were further used to calculate the predicted inhibition constants (Ki values) of the selected compounds against a given target. Specific intermolecular interactions with the targets were further visualized using BIOVIA Discovery Studio Visualizer v.4.5 (Accelrys).

### 4.3. Molecular Dynamics (MD) Simulations

For the MD simulations of the protein–drug complex, the Gromacs 5.1.4 software package was used. The lowest energy structures for the protein–ligand complex (as obtained from molecular docking) were used as the starting structure for MD simulations. AmberTools12 was used to parameterize the *Z*-guggulsterone molecule in the framework of the general Amber force field (GAFF) and AM1-BCC charges. The AMBER99SB-ILDN force field format was selected for the topology parameters of proteins. The complex was solvated in a cubical box with explicit water molecules. Periodic boundary conditions and the TIP3P water model were used. For neutralization of the system, Na^+^ ions were added. To remove steric clashes and for optimization, the steepest descent energy minimization method was used. Two-phase equilibration was performed: a 100 ps (canonical) NVT equilibration and 100 ps (thermal-isobaric) NPT equilibration. The LINCS algorithm was used to apply the position-restrained dynamics to the complex. MD simulation was performed for 50 ns and snapshots were taken every 10 ps. The Verlet algorithm was used (coupling constant of 0.1 ps) and the temperature was kept constant at 300 K. Standard pressure (1 bar) was maintained with a Parrinello–Rahman barostat (coupling constant of 2 ps). For the integration of equations of motion, a time step of 2 fs was used. For Lennard-Jones and Coulomb interactions, the cut-off distance was 1.4 nm and the electrostatic interactions were calculated using the particle mesh Ewald (PME) method. For Fourier transformation, the grid spacing was set at 0.16 nm. LigPlot was used for the analysis of hydrophobic interactions and hydrogen bonding between the protein and ligand. 

### 4.4. Free Binding Energy Calculations

Computation of ligand-free binding energies was performed using the MM/PBSA method (g_mmpbsa in the GROMACS tool). This feature analyzes all parameters of the energy for the protein and ligand in an aqueous solvent and, therefore, gives an idea of the free energy of binding of the ligand associated with the protein. To calculate the free binding energy for *Z*-guggulsterone complexed with the selected proteins, coordinates saved from the last 10 ns (41–50 ns) of the MD simulation runs were used. The binding free energy (ΔG_bind_) for the protein–ligand complex in aqueous solvent was calculated using the following equation:

ΔG_bind_ = G_complex_ − (G_protein_ + G_ligand_) (2)

where G_complex_ denotes the energy of the protein–ligand complex, and G_protein_ and G_ligand_ are the energies of the protein and ligand, respectively. The free energy for each of the above was calculated as follows:

G_x_ = E_bonded_ + (E_vdw_ + E_elec_) + G_polar_ + γSASA + b (3)

where G_x_ can be G_ligand_, G_protein_ or G_complex_; E_bonded_ denotes the energy contributed by bonded interactions and is always equal to zero; E_vdw_ represents the van der Waals energy; and E_elec_ denotes the electrostatic energy. G_polar_ is the electrostatic solvation free energy and G_non-polar_ is the apolar solvation free energy. SASA is the solvent-accessible surface area, γ is a surface tension coefficient and b is the fitting parameter. 

### 4.5. Cell Culture

The human hepatoma (HepG2, Hep3B, and Huh7) cell lines were purchased from the National Centre for Cell Science (NCCS), Pune, India. Primary hepatocytes were procured from American Type Culture Collection (ATCC), USA. HepG2, Hep3B and Huh7 cells were grown in culture flasks (Nunclon, Denmark) and maintained in DMEM supplemented with 10% FBS and 1% PenStrep solution in a humidified 5% CO_2_ incubator at 37 °C. For the cultivation of primary hepatocyte (THLE-2) cells, DMEM/F-12 media were used. The final growth medium consisted of the following: DMEM/F-12 with 10% FBS, 5 ng/mL EGF and 70 ng/mL phosphoethanolamine. THLE-2 cells require a special coating medium, which consists of the following: DMEM/F-12 without glutamine supplemented with BSA (heat shock fraction) and type I collagen peptide. Before seeding the coating, the medium was aspirated. Sub-confluent cells were harvested using 0.25% trypsin–EDTA and cells were re-suspended in complete media and counted using a haemocytometer. Each experimental data point represents the mean of triplicate wells from three independent experiments.

#### 4.5.1. MTT Assay

The MTT assay was performed to evaluate the viability of cells. A total of 1 × 10^4^ cells were seeded in a 96-well plate and allowed to adhere for 24 h. Active constituents from DuK (*Z*-guggulsterone (12.5–100 μM), dehydrocostus lactone (2–16 μM) and crocin (0.4–12.8 mg/mL)) were diluted in culture medium and added to the wells. Cells with no added treatment were used as a control. 24 h after treatment, a working solution of MTT (0.5 mg/mL) was added and incubated for 4 h at 37 °C. The MTT was discarded and DMSO was added in the dark, followed by incubation of 15 min. Regorafenib (1–8 μM) was used as a positive control. Formazan was quantified at 570 nm using a NanoDrop (Wilmington, DE, USA). The cellular morphological changes were observed under ×10 magnification (Nikon Eclipse Ti-U inverted microscope, Nikon Instruments Inc., Melville, NY, USA) using advanced research software (Nikon NIS).

#### 4.5.2. Scratch Test Assay

Cells were seeded in 6-well plates and cultured (37 °C) until 100% confluence, then scratched using a 200 μL pipette tip when the cells covered the well. The cells were washed with PBS twice to clear the floating cells and the sub-IC_50_ concentration of the active constituents of Duk was added. Images were captured at 0 and 24 h under ×10 magnification (Nikon Eclipse Ti-U inverted microscope) using advanced research software (Nikon NIS). The pictures were evaluated using Image J 1.52a software (Wayne Rasband, National Institute of Health, Bethesda, MD, USA). The data were recorded and analyzed using the following equation:

wound closure (%) = (area at hour 0 − area at hour n)/area at hour 0 × 100(4)

### 4.6. Colony Formation Assay

Huh7 cells were seeded into 6-well plates at a density of 800 cells/well and incubated for 24 h. After 24 h, the cells were treated with IC_30_, IC_40_ and IC_50_ values of *Z*-guggulsterone or a vehicle alone. Cells were incubated for an additional 8 days to allow colonies to form. After incubation, the cells were fixed with 100% methanol for 30 min at room temperature, and the plates were air-dried. The colonies were stained with 1% Coomassie blue stain, washed to remove the excess dye and imaged. The data are presented as percentages of the control group.

### 4.7. Anti-Oxidant Activity of Z-Guggulsterone

#### 4.7.1. DPPH Radical Scavenging Assay

A 0.1 mM methanolic solution of DPPH was prepared and added to different concentrations (0.25–8 μM) of *Z*-guggulsterone. Each mixture was vortexed and allowed to rest in the dark at room temperature for 30 min. The decrease in the absorbance was measured with spectrophotometric monitoring at 517 nm. Ascorbic acid was used as a positive control. Percentage inhibition of the DPPH radical was calculated using the following equation:

% DPPH radical scavenging activity = {(OD_control_ − OD_sample_)/OD_control_} × 100 (5)

where OD_control_ is the absorbance of the control and OD_sample_ is the absorbance of the *Z*-guggulsterone/ascorbic acid. Percentage inhibition was plotted against concentration. All experiments were performed in triplicate.

#### 4.7.2. Ferrous Reducing Antioxidant Power (FRAP) Assay

One milliliter of solution containing different concentrations (320–1280 μM) of *Z*-guggulsterone, 2.5 mL of potassium buffer (0.2 M) and 2.5 mL of 1% potassium ferricyanide solution were added into test tubes. The reaction mixtures were incubated for 20 min at 50 °C to complete the reaction. After incubation, 2.5 mL of 10% trichloroacetic acid solution was added to the test tubes. Each mixture was centrifuged at 700× *g* for 10 min. Then, 2.5 mL of supernatant was withdrawn and 2.5 mL of distilled water and 0.5 mL of 0.1% ferric chloride solution were added to it. The solution mixture without *Z*-guggulsterone was treated as a blank. The absorbance of the solution was measured at 700 nm using a spectrophotometer against the blank. The experiment was repeated three times at each concentration.

### 4.8. Gene Expression Analysis by qRTPCR

Huh7 cells were cultured in 60 mm dishes and treated with a sub-IC_50_ concentration of *Z*-guggulsterone for 24 h. Cells were washed with PBS and extracted with TRI reagent. Total RNA was isolated, following the protocol of Sarwat and Naqvi [40]. RNA was dissolved in 30 μL nuclease-free water. The isolated RNAs were analyzed by electrophoresis on a 1% agarose gel. RNA was quantified with a NanoDrop (NanoDrop Technologies, Wilmington, DE, USA) and the concentration of RNA was calculated from the optical density at 260 nm. The purity of RNA was determined using 260 nm/280 nm of absorbance. Further, cDNA was synthesized from total RNA templates using a High Capacity cDNA kit. RNA was reverse transcribed using 1000 ng of total RNA and 10X RT primers following the manufacturer’s instructions. The cDNA synthesized was stored at −20 °C for later use as a template for qRT-PCR.

To study the expression profiles of the selected genes (based on in silico studies), quantitative real-time PCR was used on the untreated control and the cells treated with a sub-IC_50_ concentration of *Z*-guggulsterone. In each reaction mixture, 1μL (~50 ng) cDNA was used with gene-specific forward and reverse primers (Supplementary Materials Table S4), along with the SYBR Green master mix. A Step OneTM Real-Time PCR System (Applied Biosystems, Foster City, CA, USA) was used for the assessment of the samples. Along with the gene of interest, β-actin was run as a reference control to normalize gene expression. The reaction procedure utilized a duration of 10 min at 95 °C, followed by 45 cycles at 95 °C (15 s) and 60 °C for 1 min. All the experiments were performed in triplicate, along with negative and positive controls. The expression fold change was calculated with reference to the normal control group based on the threshold cycle (CT) values by using the following formula:

relative quantification (RQ) = 2 − ∆∆CT(6)

### 4.9. Cell Cycle Analysis

Huh7 cells (1 × 10^6^ /well) cells were cultured in 6-well plates in the presence of *Z*-guggulsterone (0, 10 and 18 μM) for 24 h. After treatment, cells were harvested and fixed with 70% ethanol at 4 °C for 2 h. After fixing, the cells were washed twice with PBS and centrifuged at 850× *g* for 5 min at 4 °C. The pellet was broken up by vortexing and then re-suspended in PBS containing 100 μg/mL RNase A and 50 μg/mL propidium iodide (PI) and incubated for a further 30 min in the dark. Finally, the cells were analyzed using flow cytometry (BD FACS Calibur, BD Biosciences, San Jose, CA, USA).

### 4.10. Statistical Analysis

All experimental results are from at least three identical experiments and expressed as means ± standard deviation. Data were analyzed using GraphPad (version 5.01, San Diego, CA, USA) and compared using regression analysis and two-way ANOVA. Differences between control and treatment groups were determined at a significance level of *p* > 0.05.

## 5. Conclusions and Future Perspectives

It was initially assumed that saffron is the major herb that is responsible for the pleiotropic activities of DuK. We used an integrated approach involving molecular docking, dynamics simulation and in vitro studies and gained structural and mechanistic insights about the possible lead molecule amongst the drug-like bioactive compounds of DuK. In silico, *Z*-guggulsterone was predicted to interact better than crocin and other selected constituents with the key molecular targets of HCC and ER stress, and it inhibited the viability of human HCC cells by inhibiting clone-forming potential and regulating the cell cycle. However, more studies at the in vitro and in vivo levels are necessary to understand the exact role and mechanism involved. Subsequent studies could be conducted on downstream pathways associated with *ezrin*, *calreticulin*, *aminoacylase 1* and RB1. Further studies will also be needed to investigate the possible contributions of the other constituents of DuK, which may act in an additive or even synergistic manner through different mechanisms. 

## Figures and Tables

**Table 1:** Predicted binding interactions of the best three (out of ten) active constituents and the best standard drug (out of three) with target proteins involved in hepatocellular carcinoma (HCC) and ER stress.

S. No.	Proteins Related to HCC/ER Stress	Dehydrocostus Lactone		*Z*-Guggulsterone		Crocin		Regorafenib	
		BA	Ki (μM)	BA	Ki (μM)	BA	Ki (μM)	BA	Ki (μM)
1.	Cyclin E1	−6.8	10.3641	−7.5	3.17997	−7.0	7.39482	−8.0	1.36747
2.	Transgelin	−6.6	14.5255	−7.7	2.26893	−5.7	66.35	−7.6	2.6861
3.	S100A14	−8.1	1.15509	−8.1	1.15509	−5.8	56.0454	−8.0	1.36747
4.	Prohibitin	−5.7	66.35	−6.5	17.1962	−5.5	92.9915	−6.4	20.3579
5.	Ezrin	−7.0	7.39482	−8.7	0.419575	−7.3	4.45681	−8.4	0.696166
6.	Retinoblastoma protein (RB1)	−7.0	7.39482	−7.7	2.26893	−6.6	14.5255	−8.3	0.824165
7.	Axis inhibition protein 1 (AXIN1)	−6.0	39.9888	−8.4	0.696166	−5.2	154.293	−7.4	3.76464
8.	Aminoacylase 1	−6.8	10.3641	−7.7	2.26893	−7.3	4.45681	−8.4	0.696166
9.	P58	−6.4	20.3579	−7.2	5.27625	−6.7	12.2696	−7.2	5.27625
10.	Calreticulin	−6.4	20.3579	−8.2	0.975697	−6.5	17.1962	−7.5	3.17997
11.	Protein disulfide isomerase (PDI)	−7.3	4.45681	−8.3	0.824165	−7.7	2.26893	−8.5	0.588047

**Table 2:** MMPBSA binding energy for the protein–drug complexes from the last 10 ns of the simulation.

Name of the Protein/PDB ID	vdW	Electro	Polar S	SASA	WCA	BE
1ad6/RB1	−122.089	4.856	51.879	−13.668	67.669	−11.353
1ni2/ezrin	−88.838	−17.231	46.024	−10.412	53.422	−17.034
1q7l/aminoacyclase 1	−136.432	−16.718	65.543	−12.560	0.00	−100.169
1w98/cyclin E1	−101.134	5.898	51.369	−11.193	59.079	4.020
5lk5/Calreticulin	−72.960	−22.837	34.162	−8.369	61.288	−8.7171

## Data Availability

The data generated from the study are clearly presented and discussed in the manuscript.

## Supplementary Materials

The following are available online at https://www.mdpi.com/article/10.3390/molecules27165104/s1, Figure S1. Two-dimensional (2D) interaction diagram (left) and three-dimensional (3D) molecular dynamics simulation poses (right) for Z-guggulsterone complexed with selected proteins: cyclin E1, ezrin, RB1, aminoacylase, transgelin, S100A14, prohibitin, AXIN1, PDI, CRT and P58; Figure S2. Chemical structure of the bioactive components of DuK; Table S1. Predicted binding affinities (docking scores) and inhibition constants of the 10 selected active constituents of Dawa-ul-Kurkum against the target proteins involved in different stages of hepatocellular carcinoma. For each protein, the active constituents are tabulated in descending order of binding affinity; Table S2. Predicted binding affinities (docking scores) and inhibition constants of the 10 selected active constituents of Dawa-ul-Kurkum against the target proteins involved in endoplasmic reticulum stress. For each protein, the active constituents are tabulated in descending order of binding affinity; Table S3. Predicted binding affinities (docking scores) and inhibition constants of the three reference drugs against the target proteins involved in different stages of hepatocellular carcinoma and endoplasmic reticulum stress. For each protein, the reference drugs are tabulated in descending order of binding affinity; Table S4. Primer sequences for qRT-PCR.

## Abbreviations

American Type Culture Collection	ATCC
bovine serum albumin	BSA
Dawa-ul-Kurkum	DuK
deoxyribonucleic Acid	DNA
2,2-diphenyl-1-picrylhydrazyl	DPPH
epithelial-to-mesenchymal transition	EMT
ezrin–radixin–moesin	ERM
ferrous reducing antioxidant power	FRAP
hepatitis B/C virus	HBV/HCV
hepatocellular carcinoma	HCC
hepatoma G2	HepG2
hepatoma 3B	Hep3B
human hepatoma-derived-7	HuH-7
molecular dynamics	MD
National Centre for Cell Science	NCCS
quantitative reverse transcriptase polymerase chain reaction	qRT-PCR
relative quantification	RQ
ribonucleic acid	RNA
