# Supplementary material for: Z-Guggulsterone Is a Potential Lead Molecule of Dawa-ul-Kurkum against Hepatocellular Carcinoma
Source: Molecules. 2022 Aug 11;27(16):5104. doi: 10.3390/molecules27165104 (PMC9413334; doi:10.3390/molecules27165104)
Supplement: Supplementary file 1 [file molecules-27-05104-s001.zip › molecules-1785524-supplementary.pdf]

Table S1: Predicted binding affinity (docking scores) and inhibition constant of the ten selected active constituents of Dawa-ul-Kurkum against the target proteins involved in different stages of hepatocellular carcinoma. For each protein, the active constituents are tabulated in descending order of binding affinity.

S. No.	Proteins Involved/ PDB ID	Biological activity/Pathway	Active constituents	Name of the plant	Binding affinity (Kcal/Mol)	Inhibition constant, Ki (µM)
1.	Cyclin E1/1W98	Cell cycle regulator	Z-Guggulsterone	*Commiphora wightii*	-7.5	3.17997
			Crocin	*Crocus sativus*	-7.0	7.39482
			Dehydrocostus lactone	*Saussurea lappa*	-6.8	10.3641
			Jatamansone	*Nardostachys jatamansi*	-6.7	12.2696
			Crocetin	*Crocus sativus*	-6.5	17.1962
			p-Coumaric acid	*Cinnamomum cassia*	-5.0	216.246
			Citral	*Cymbopogon jwarancusa*	-5.0	216.246
			Safranal	*Crocus sativus*	-5.0	216.246
			Cinnamaldehyde	*Cinnamomum cassia & zeylanicum*	-4.8	303.075
			Isovaleric acid	*Nardostachys jatamansi*	-3.8	1638.931
2.	Cyclin D1 (CCND1)/ 2W9F	Tumor differentiation	Z-Guggulsterone	*Commiphora wightii*	-7.4	3.76464
			Dehydrocostus lactone	*Saussurea lappa*	-7.2	5.27625
			Crocin	*Crocus sativus*	-6.4	20.3579
			Crocetin	*Crocus sativus*	-6.3	24.101
			p-Coumaric acid	*Cinnamomum cassia*	-6.2	28.5322
			Jatamansone	*Nardostachys jatamansi*	-6.0	39.9888

			Safranal	*Crocus sativus*	-5.4	110.089
			Cinnamaldehyde	*Cinnamomum cassia & zeylanicum*	-5.3	130.33
			Citral	*Cymbopogon jwarancusa*	-4.8	303.075
			Isovaleric acid	*Nardostachys jatamansi*	-3.6	2297.009
3.	Fibronectin/ 1FNF	Provokes late stages of cancer metastasis	Z-Guggulsterone	*Commiphora wightii*	-7.3	4.45681
			Crocin	*Crocus sativus*	-6.3	24.101
			Dehydrocostus lactone	*Saussurea lappa*	-6.2	28.5322
			Crocetin	*Crocus sativus*	-5.9	47.3412
			Jatamansone	*Nardostachys jatamansi*	-5.7	66.35
			p-Coumaric acid	*Cinnamomum cassia*	-4.9	256.006
			Safranal	*Crocus sativus*	-4.7	358.799
			Citral	*Cymbopogon jwarancusa*	-4.5	502.867
			Cinnamaldehyde	*Cinnamomum cassia & zeylanicum*	-4.4	595.325
			Isovaleric acid	*Nardostachys jatamansi*	-4.1	987.774

4.	Peroxiredoxin 2 (PRDX2)/ 1QMV	ROS reduction	Z-Guggulsterone	*Commiphora wightii*	-7.3	4.45681
			Crocin	*Crocus sativus*	-6.4	20.3579
			Crocetin	*Crocus sativus*	-6.2	28.5322
			Dehydrocostus lactone	*Saussurea lappa*	-6.2	28.5322
			Jatamansone	*Nardostachys jatamansi*	-5.8	56.0454
			p-Coumaric acid	*Cinnamomum cassia*	-5.2	154.293
			Safranal	*Crocus sativus*	-4.6	424.769
			Cinnamaldehyde	*Cinnamomum cassia & zeylanicum*	-4.6	424.769
			Citral	*Cymbopogon jwarancusa*	-4.4	595.325
			Isovaleric acid	*Nardostachys jatamansi*	-4.0	1169.388
5.	Transgelin/ 1WYM	NF-κB signalling	Z-Guggulsterone	*Commiphora wightii*	-7.7	2.26893
			Crocetin	*Crocus sativus*	-6.8	10.3641
			Dehydrocostus lactone	*Saussurea lappa*	-6.6	14.5255
			Jatamansone	*Nardostachys jatamansi*	-6.2	28.5322
			Citral	*Cymbopogon jwarancusa*	-5.8	56.0454
			p-Coumaric acid	*Cinnamomum cassia*	-5.8	56.0454
			Cinnamaldehyde	*Cinnamomum cassia & zeylanicum*	-5.8	56.0454
			Crocin	*Crocus sativus*	-5.7	66.35

			Safranal	*Crocus sativus*	-5.2	154.293
			Isovaleric acid	*Nardostachys jatamansi*	-4.4	595.325
6.	Protein disulfide-isomerase A3 (PDIA3)/ 2DMM	Increase in cancer cell proliferation and a reduction in apoptotic cell death	Z-Guggulsterone	*Commiphora wightii*	-7.3	4.45681
			Crocin	*Crocus sativus*	-6.6	14.5255
			Dehydrocostus lactone	*Saussurea lappa*	-6.2	28.5322
			Jatamansone	*Nardostachys jatamansi*	-5.8	56.0454
			Crocetin	*Crocus sativus*	-5.8	56.0454
			p-Coumaric acid	*Cinnamomum cassia*	-5.1	182.662
			Safranal	*Crocus sativus*	-4.8	303.075
			Cinnamaldehyde	*Cinnamomum cassia & zeylanicum*	-4.4	595.325
			Citral	*Cymbopogon jwarancusa*	-4.2	834.366
			Isovaleric acid	*Nardostachys jatamansi*	-4.0	1169.388
	2H8L		Z-Guggulsterone	*Commiphora wightii*	-7.4	3.76464
			Dehydrocostus lactone	*Saussurea lappa*	-6.6	14.5255
			Crocin	*Crocus sativus*	-6.4	20.357
			Crocetin	*Crocus sativus*	-6.3	24.101
			Cinnamaldehyde	*Cinnamomum cassia & zeylanicum*	-5.6	78.5493
			Jatamansone	*Nardostachys jatamansi*	-5.4	110.089

			p-Coumaric acid	*Cinnamomum cassia*	-5.2	154.293
			Safranal	*Crocus sativus*	-5.0	216.246
			Isovaleric acid	*Nardostachys jatamansi*	-4.5	502.867
			Citral	*Cymbopogon jwarancusa*	-4.2	834.366
7.	Insulin-like growth factor 2 (IGF2)/ 2H7T	Receptor tyrosine kinase pathway	Z-Guggulsterone	*Commiphora wightii*	-7.7	2.26893
			Dehydrocostus lactone	*Saussurea lappa*	-6.8	10.3641
			Crocin	*Crocus sativus*	-6.5	17.1962
			Crocetin	*Crocus sativus*	-6.4	20.3579
			Jatamansone	*Nardostachys jatamansi*	-5.9	47.3412
			Safranal	*Crocus sativus*	-5.1	182.662
			p-Coumaric acid	*Cinnamomum cassia*	-5.0	216.246
			Cinnamaldehyde	*Cinnamomum cassia & zeylanicum*	-4.5	502.867
			Citral	*Cymbopogon jwarancusa*	-4.5	502.867
			Isovaleric acid	*Nardostachys jatamansi*	-3.7	1940.268
8.	S100A14/ 2M0R	RAF/ERK/MAPK pathway	Z-Guggulsterone	*Commiphora wightii*	-8.1	1.15509
			Dehydrocostus lactone	*Saussurea lappa*	-8.1	1.15509
			Crocetin	*Crocus sativus*	-6.5	17.1962
			Jatamansone	*Nardostachys jatamansi*	-6.3	24.101
			Safranal	*Crocus sativus*	-6.0	39.9888

			Crocin	*Crocus sativus*	-5.8	56.0454
			Citral	*Cymbopogon jwarancusa*	-5.8	56.0454
			p-Coumaric acid	*Cinnamomum cassia*	-5.4	110.089
			Cinnamaldehyde	*Cinnamomum cassia & zeylanicum*	-5.3	130.33
			Isovaleric acid	*Nardostachys jatamansi*	-4.2	834.366
9.	Cyclin-dependent kinase 4 (CDK4)/ 2W99	TGF-β signalling	Z-Guggulsterone	*Commiphora wightii*	-8.2	0.975697
			Dehydrocostus lactone	*Saussurea lappa*	-8.2	0.975697
			Crocetin	*Crocus sativus*	-7.3	4.45681
			Jatamansone	*Nardostachys jatamansi*	-7.3	4.45681
			Crocin	*Crocus sativus*	-7.0	7.39482
			p-Coumaric acid	*Cinnamomum cassia*	-6.2	28.5322
			Safranal	*Crocus sativus*	-6.2	28.5322
			Cinnamaldehyde	*Cinnamomum cassia & zeylanicum*	-5.4	110.089
			Citral	*Cymbopogon jwarancusa*	-5.3	130.33
			Isovaleric acid	*Nardostachys jatamansi*	-4.2	834.366
10.	Spondin-2/ 3D34	Wnt/β-catenin pathway	Z-Guggulsterone	*Commiphora wightii*	-7.3	4.45681
			Dehydrocostus lactone	*Saussurea lappa*	-6.7	12.2696
			Jatamansone	*Nardostachys jatamansi*	-6.3	24.101

			Crocin	*Crocus sativus*	-6.0	39.9888
			Crocetin	*Crocus sativus*	-5.9	47.3412
			p-Coumaric acid	*Cinnamomum cassia*	-5.2	154.293
			Safranal	*Crocus sativus*	-5.2	154.293
			Citral	*Cymbopogon jwarancusa*	-4.8	303.075
			Cinnamaldehyde	*Cinnamomum cassia & zeylanicum*	-4.5	502.867
			Isovaleric acid	*Nardostachys jatamansi*	-3.9	1384.394
11.	Bone marrow stromal antigen 2 (BST2)/ 3NWH	pERK pathway; NF-κB signalling	Z-Guggulsterone	*Commiphora wightii*	-5.7	6.635E-05
			Dehydrocostus lactone	*Saussurea lappa*	-5.4	110.089
			Crocetin	*Crocus sativus*	-5.3	130.33
			Crocin	*Crocus sativus*	-4.4	595.325
			Jatamansone	*Nardostachys jatamansi*	-4.2	834.366
			p-Coumaric acid	*Cinnamomum cassia*	-4.0	1169.388
			Safranal	*Crocus sativus*	-4.0	1169.388
			Citral	*Cymbopogon jwarancusa*	-3.7	1940.268
			Cinnamaldehyde	*Cinnamomum cassia & zeylanicum*	-3.5	2719.342
			Isovaleric acid	*Nardostachys jatamansi*	-3.1	5341.558
12.	C-reactive protein (CRP)/ 3PVN	Progression of tumor-promoting inflammation	Z-Guggulsterone	*Commiphora wightii*	-7.1	6.24635
			Dehydrocostus lactone	*Saussurea lappa*	-7.1	6.24635

			Jatamansone	*Nardostachys jatamansi*	-6.5	17.1962
			Crocetin	*Crocus sativus*	-6.3	24.101
			Crocin	*Crocus sativus*	-6.1	33.7782
			p-Coumaric acid	*Cinnamomum cassia*	-5.5	92.9915
			Safranal	*Crocus sativus*	-5.1	182.662
			Citral	*Cymbopogon jwarancusa*	-4.8	303.075
			Cinnamaldehyde	*Cinnamomum cassia & zeylanicum*	-4.7	358.799
			Isovaleric acid	*Nardostachys jatamansi*	-3.9	1384.394
13.	Peroxiredoxin 6/ 5B6M	Ras and JAK/STAT pathway	Z-Guggulsterone	*Commiphora wightii*	-7.4	3.76464
			Crocetin	*Crocus sativus*	-6.5	17.1962
			Dehydrocostus lactone	*Saussurea lappa*	-6.2	28.5322
			Crocin	*Crocus sativus*	-6.0	39.9888
			Jatamansone	*Nardostachys jatamansi*	-5.9	47.3412
			p-Coumaric acid	*Cinnamomum cassia*	-5.1	182.662
			Safranal	*Crocus sativus*	-4.6	424.769
			Cinnamaldehyde	*Cinnamomum cassia & zeylanicum*	-4.5	502.867
			Citral	*Cymbopogon jwarancusa*	-4.3	704.783
			Isovaleric acid	*Nardostachys jatamansi*	-3.8	1638.931
14.	Prohibitin/ 6IQE	Proliferation, metastasis and	Z-Guggulsterone	*Commiphora wightii*	-6.5	17.1962

		apoptosis				
			Crocetin	*Crocus sativus*	-5.8	56.0454
			Dehydrocostus lactone	*Saussurea lappa*	-5.7	66.35
			Crocin	*Crocus sativus*	-5.5	92.9915
			Jatamansone	*Nardostachys jatamansi*	-5.4	110.089
			Isovaleric acid	*Nardostachys jatamansi*	-4.3	704.783
			Citral	*Cymbopogon jwarancusa*	-4.1	987.774
			p-Coumaric acid	*Cinnamomum cassia*	-4.0	1169.388
			Cinnamaldehyde	*Cinnamomum cassia & zeylanicum*	-3.9	1384.394
			Safranal	*Crocus sativus*	-3.9	1384.394
15.	C-X-C motif chemokine ligand 10 (CXCL10)/ 1O7Y	Migration and invasion	Z-Guggulsterone	*Commiphora wightii*	-5.9	47.3412
			Crocetin	*Crocus sativus*	-5.5	92.9915
			Dehydrocostus lactone	*Saussurea lappa*	-5.4	110.089
			Crocin	*Crocus sativus*	-4.9	256.006
			Jatamansone	*Nardostachys jatamansi*	-4.7	358.799
			Cinnamaldehyde	*Cinnamomum cassia & zeylanicum*	-4.1	987.774
			p-Coumaric acid	*Cinnamomum cassia*	-4.1	987.774
			Safranal	*Crocus sativus*	-4.0	1169.388
			Citral	*Cymbopogon jwarancusa*	-3.9	1384.394
16.	Ezrin/	Invasion,	Z-Guggulsterone	*Commiphora*	-8.7	0.419575

	1NI2	migration and poor prognosis		*wightii*		
			Crocin	*Crocus sativus*	-7.3	4.45681
			Dehydrocostus lactone	*Saussurea lappa*	-7.0	7.39482
			Crocetin	*Crocus sativus*	-6.5	17.1962
			Jatamansone	*Nardostachys jatamansi*	-6.4	20.3579
			p-Coumaric acid	*Cinnamomum cassia*	-5.7	66.35
			Safranal	*Crocus sativus*	-5.3	130.33
			Citral	*Cymbopogon jwarancusa*	-5.1	182.662
			Cinnamaldehyde	*Cinnamomum cassia & zeylanicum*	-5.0	216.246
			Isovaleric acid	*Nardostachys jatamansi*	-4.3	704.783
17.	Retinoblastoma protein (RB1)/ 1AD6	Cell cycle modulator	Z-Guggulsterone	*Commiphora wightii*	-7.7	2.26893
			Dehydrocostus lactone	*Saussurea lappa*	-7.0	7.39482
			Crocin	*Crocus sativus*	-6.6	14.5255
			Crocetin	*Crocus sativus*	-6.5	17.1962
			Jatamansone	*Nardostachys jatamansi*	-6.4	20.3579
			p-Coumaric acid	*Cinnamomum cassia*	-5.4	110.089
			Safranal	*Crocus sativus*	-5.2	154.293
			Cinnamaldehyde	*Cinnamomum cassia & zeylanicum*	-4.8	303.075
			Citral	*Cymbopogon jwarancusa*	-4.7	358.799

			Isovaleric acid	*Nardostachys jatamansi*	-3.9	1384.394
18.	Axis inhibition protein 1 (AXIN1)/ 1DK8	WNT/β-catenin pathway	Z-Guggulsterone	*Commiphora wightii*	-8.4	0.696166
			Dehydrocostus lactone	*Saussurea lappa*	-6.0	39.9888
			Jatamansone	*Nardostachys jatamansi*	-5.9	47.3412
			Crocetin	*Crocus sativus*	-5.8	56.0454
			Crocin	*Crocus sativus*	-5.2	154.293
			p-Coumaric acid	*Cinnamomum cassia*	-5.2	154.293
			Safranal	*Crocus sativus*	-4.7	358.799
			Cinnamaldehyde	*Cinnamomum cassia & zeylanicum*	-4.6	424.769
			Citral	*Cymbopogon jwarancusa*	-4.3	704.783
			Isovaleric acid	*Nardostachys jatamansi*	-3.8	1638.931
19.	Insulin like growth factor 2 receptor (IGF2R)/ 1GP0	TGF-β-mediated growth control	Z-Guggulsterone	*Commiphora wightii*	-6.8	10.3641
			Crocetin	*Crocus sativus*	-6.5	17.1962
			Dehydrocostus lactone	*Saussurea lappa*	-6.0	39.9888
			Jatamansone	*Nardostachys jatamansi*	-5.8	56.0454
			Crocin	*Crocus sativus*	-4.8	303.075
			Citral	*Cymbopogon jwarancusa*	-4.8	303.075
			p-Coumaric acid	*Cinnamomum cassia*	-4.7	358.799
			Safranal	*Crocus sativus*	-4.5	502.867

			Cinnamaldehyde	*Cinnamomum cassia & zeylanicum*	-4.5	502.867
			Isovaleric acid	*Nardostachys jatamansi*	-3.7	1940.268
20.	SMAD Family Member 2 (SMAD2)/ 1KHX	TGF-β-mediated growth control	Dehydrocostus lactone	*Saussurea lappa*	-7.4	3.76464
			Z-Guggulsterone	*Commiphora wightii*	-7.4	3.76464
			Jatamansone	*Nardostachys jatamansi*	-6.5	17.1962
			Crocetin	*Crocus sativus*	-6.4	20.3579
			Crocin	*Crocus sativus*	-6.1	33.7782
			Safranal	*Crocus sativus*	-5.9	47.3412
			p-Coumaric acid	*Cinnamomum cassia*	-5.7	66.35
			Cinnamaldehyde	*Cinnamomum cassia & zeylanicum*	-5.5	92.9915
			Citral	*Cymbopogon jwarancusa*	-5.2	154.293
			Isovaleric acid	*Nardostachys jatamansi*	-4.5	502.867
21.	Aminoacylase 1/1Q7L	TGF-β1 and ERK1 pathway	Z-Guggulsterone	*Commiphora wightii*	-7.7	2.26893
			Crocin	*Crocus sativus*	-7.3	4.45681
			Crocetin	*Crocus sativus*	-6.8	10.3641
			Dehydrocostus lactone	*Saussurea lappa*	-6.8	10.3641
			Jatamansone	*Nardostachys jatamansi*	-6.0	39.9888
			Citral	*Cymbopogon jwarancusa*	-4.9	256.006

			p-Coumaric acid	*Cinnamomum cassia*	-5.2	154.293
			Safranal	*Crocus sativus*	-5.0	216.246
			Cinnamaldehyde	*Cinnamomum cassia & zeylanicum*	-4.6	424.769
			Isovaleric acid	*Nardostachys jatamansi*	-3.7	1940.268
22.	Tumor protein p53 (TP53)/ 1TUP	Cell cycle regulator; Ubiquitin-proteasome pathway	Z-Guggulsterone	*Commiphora wightii*	-6.9	8.75444
			Crocin	*Crocus sativus*	-6.0	39.9888
			Crocetin	*Crocus sativus*	-6.0	39.9888
			Dehydrocostus lactone	*Saussurea lappa*	-6.0	39.9888
			Jatamansone	*Nardostachys jatamansi*	-5.3	130.33
			p-Coumaric acid	*Cinnamomum cassia*	-4.8	303.075
			Safranal	*Crocus sativus*	-4.6	424.769
			Cinnamaldehyde	*Cinnamomum cassia & zeylanicum*	-4.4	595.325
			Isovaleric acid	*Nardostachys jatamansi*	-4.4	595.325
			Citral	*Cymbopogon jwarancusa*	-4.0	1169.388

Table S2: Predicted binding affinity (docking scores) and inhibition constant of the ten selected active constituents of Dawa-ul-Kurkum against the target proteins involved in endoplasmic reticulum stress. For each protein, the active constituents are tabulated in descending order of binding affinity.

S. No.	Proteins Involved/PDB ID	Biological activity/Pathway	Active constituents	Name of the plant	Binding affinity (Kcal/Mol)	Inhibition constant, Ki (µM)
1.	Activating transcription factor 4 (ATF-4)/ 1CI6	Signalling through PERK pathway	Z-Guggulsterone	*Commiphora wightii*	-6.0	39.9888
			Dehydrocostus lactone	*Saussurea lappa*	-5.5	92.9915
			Crocetin	*Crocus sativus*	-5.3	130.33
			Crocin	*Crocus sativus*	-4.8	303.075
			Jatamansone	*Nardostachys jatamansi*	-4.5	502.867
			Citral	*Cymbopogon jwarancusa*	-4.0	1169.388
			p-Coumaric acid	*Cinnamomum cassia*	-3.9	1384.394
			Safranal	*Crocus sativus*	-3.8	1638.931
			Cinnamaldehyde	*Cinnamomum cassia & zeylanicum*	-3.6	2297.009
			Isovaleric acid	*Nardostachys jatamansi*	-3.2	4511.977
2.	Death receptor 5 (DR5)/ 1D0G	Signalling through PERK	Z-Guggulsterone	*Commiphora wightii*	-6.3	24.101
			Crocin	*Crocus sativus*	-6.3	24.101
			Dehydrocostus lactone	*Saussurea lappa*	-5.8	56.0454
			Crocetin	*Crocus sativus*	-5.5	92.9915
			Jatamansone	*Nardostachys jatamansi*	-5.0	216.246
			p-Coumaric acid	*Cinnamomum cassia*	-4.8	303.075

			Safranal<br>Citral	*Crocus sativus*<br>*Cymbopogon jwarancusa*	-4.1<br>-4.0	987.774<br>1169.388
			Cinnamaldehyde	*Cinnamomum cassia & zeylanicum*	-3.9	1384.394
			Isovaleric acid	*Nardostachys jatamansi*	-3.7	1940.268
3.	P27Kip1/ 1JSU	Signalling through PERK	Z-Guggulsterone	*Commiphora wightii*	-7.1	6.24635
			Crocetin	*Crocus sativus*	-6.2	28.5322
			Dehydrocostus lactone	*Saussurea lappa*	-6.2	28.5322
			Jatamansone	*Nardostachys jatamansi*	-5.8	56.0454
			Crocin	*Crocus sativus*	-5.6	78.5493
			Safranal	*Crocus sativus*	-4.7	358.799
			Citral	*Cymbopogon jwarancusa*	-4.5	502.867
			p-Coumaric acid	*Cinnamomum cassia*	-4.1	987.774
			Cinnamaldehyde	*Cinnamomum cassia & zeylanicum*	-3.9	1384.394
			Isovaleric acid	*Nardostachys jatamansi*	-3.5	2719.342
4.	Eukaryotic Initiation Factor 2α (eIF2α)/ 1KL9	Signalling through PERK	Z-Guggulsterone	*Commiphora wightii*	-6.9	8.75444
			Dehydrocostus lactone	*Saussurea lappa*	-6.7	12.2696
			Crocin	*Crocus sativus*	-6.7	12.2696
			Crocetin	*Crocus sativus*	-6.1	33.7782
			Jatamansone	*Nardostachys jatamansi*	-5.9	47.3412

			p-Coumaric acid	*Cinnamomum cassia*	-5.3	130.33
			Safranal	*Crocus sativus*	-5.2	154.293
			Cinnamaldehyde	*Cinnamomum cassia & zeylanicum*	-5.1	182.662
			Citral	*Cymbopogon jwarancusa*	-4.8	303.075
			Isovaleric acid	*Nardostachys jatamansi*	-4.1	987.774
5.	P58/2Y4T	Signalling through ATF6	Z-Guggulsterone	*Commiphora wightii*	-7.2	5.27625
			Jatamansone	*Nardostachys jatamansi*	-6.8	10.3641
			Crocin	*Crocus sativus*	-6.7	12.2696
			Dehydrocostus lactone	*Saussurea lappa*	-6.4	20.3579
			Crocetin	*Crocus sativus*	-6.2	28.5322
			p-Coumaric acid	*Cinnamomum cassia*	-5.7	66.35
			Safranal	*Crocus sativus*	-5.7	66.35
			Cinnamaldehyde	*Cinnamomum cassia & zeylanicum*	-5.3	130.33
			Citral	*Cymbopogon jwarancusa*	-5.1	182.662
			Isovaleric acid	*Nardostachys jatamansi*	-4.4	595.325
6.	Caspase 3/ 3KJF	Signalling through IRE1	Z-Guggulsterone	*Commiphora wightii*	-6.6	14.5255
			Crocetin	*Crocus sativus*	-6.1	33.7782
			Dehydrocostus lactone	*Saussurea lappa*	-5.8	56.0454
			Crocin	*Crocus sativus*	-5.5	92.9915

			Jatamansone	*Nardostachys jatamansi*	-5.3	130.33
			p-Coumaric acid	*Cinnamomum cassia*	-4.6	424.769
			Safranal	*Crocus sativus*	-4.2	834.366
			Citral	*Cymbopogon jwarancusa*	-4.2	834.366
			Cinnamaldehyde	*Cinnamomum cassia & zeylanicum*	-3.9	1384.394
			Isovaleric acid	*Nardostachys jatamansi*	-3.7	1940.268
7.	Growth arrest and DNA damage-inducible protein 34 (GADD34)/ 4XPN	Signalling through PERK	Z-Guggulsterone	*Commiphora wightii*	-4.7	358.799
			Crocin	*Crocus sativus*	-4.4	595.325
			Crocetin	*Crocus sativus*	-4.3	704.783
			Dehydrocostus lactone	*Saussurea lappa*	-4.1	987.774
			Jatamansone	*Nardostachys jatamansi*	-3.7	1940.268
			p-Coumaric acid	*Cinnamomum cassia*	-3.4	3219.325
			Safranal	*Crocus sativus*	-3.4	3219.325
			Citral	*Cymbopogon jwarancusa*	-3.2	4511.977
			Cinnamaldehyde	*Cinnamomum cassia & zeylanicum*	-3.1	5341.558
			Isovaleric acid	*Nardostachys jatamansi*	-2.8	8862.802
8.	Glucose regulated protein 78 (GRP78)/ 5E85	Signalling through ATF6	Z-Guggulsterone	*Commiphora wightii*	-8.1	1.15509
			Crocetin	*Crocus sativus*	-7.0	7.39482

			Dehydrocostus lactone	*Saussurea lappa*	-6.6	14.5255
			Jatamansone	*Nardostachys jatamansi*	-6.5	17.1962
			Crocin	*Crocus sativus*	-6.1	33.7782
			p-Coumaric acid	*Cinnamomum cassia*	-5.8	56.0454
			Citral	*Cymbopogon jwarancusa*	-5.7	66.35
			Safranal	*Crocus sativus*	-5.3	130.33
			Cinnamaldehyde	*Cinnamomum cassia & zeylanicum*	-4.8	303.075
			Isovaleric acid	*Nardostachys jatamansi*	-4.0	1169.388
	5EVZ		Crocin	*Crocus sativus*	-8.8	0.354412
			Z-Guggulsterone	*Commiphora wightii*	-7.7	2.26893
			Crocetin	*Crocus sativus*	-7.4	3.76464
			Dehydrocostus lactone	*Saussurea lappa*	-6.5	17.1962
			Jatamansone	*Nardostachys jatamansi*	-6.0	39.9888
			p-Coumaric acid	*Cinnamomum cassia*	-6.0	39.9888
			Citral	*Cymbopogon jwarancusa*	-5.4	110.089
			Cinnamaldehyde	*Cinnamomum cassia & zeylanicum*	-5.4	110.089
			Safranal	*Crocus sativus*	-5.3	130.33
			Isovaleric acid	*Nardostachys jatamansi*	-4.5	502.867

9.	Calreticulin/ 5LK5	Protein folding	Z-Guggulsterone	*Commiphora wightii*	-8.2	0.975697
			Crocin	*Crocus sativus*	-6.5	17.1962
			Dehydrocostus lactone	*Saussurea lappa*	-6.4	20.3579
			Jatamansone	*Nardostachys jatamansi*	-6.2	28.5322
			Crocetin	*Crocus sativus*	-6.0	39.9888
			Safranal	*Crocus sativus*	-5.5	92.9915
			p-Coumaric acid	*Cinnamomum cassia*	-5.2	154.293
			Cinnamaldehyde	*Cinnamomum cassia & zeylanicum*	-4.8	303.075
			Citral	*Cymbopogon jwarancusa*	-4.5	502.867
			Isovaleric acid	*Nardostachys jatamansi*	-4.4	595.325
10.	Protein disulfide isomerase (PDI)/ 6I7S	Signalling through PERK	Z-Guggulsterone	*Commiphora wightii*	-8.3	0.824165
			Crocin	*Crocus sativus*	-7.7	2.26893
			Dehydrocostus lactone	*Saussurea lappa*	-7.3	4.45681
			Jatamansone	*Nardostachys jatamansi*	-6.8	10.3641
			Crocetin	*Crocus sativus*	-6.8	10.3641
			Safranal	*Crocus sativus*	-6.0	39.9888
			p-Coumaric acid	*Cinnamomum cassia*	-5.6	78.5493
			Citral	*Cymbopogon jwarancusa*	-5.4	110.089
			Cinnamaldehyde	*Cinnamomum cassia &*	-5.3	130.33

			Isovaleric acid	*zeylanicum*<br><br>*Nardostachys jatamansi*	-4.3	704.783

Table S3: Predicted binding affinity (docking scores) and inhibition constant of the three reference drugs against the target proteins involved in different stages of hepatocellular carcinoma and endoplasmic reticulum stress. For each protein, the reference drugs are tabulated in descending order of binding affinity.

S. No.	Proteins Involved	PDB ID	Reference drug	Binding affinity (Kcal/Mol)	Inhibition constant, Ki (µM)
1.	Cyclin E1	1W98	Regorafenib<br>Sorafenib<br>Nivolumab	-8.0<br>-7.7<br>-7.7	1.36747<br>2.26893<br>2.26893
2.	Cyclin D1 (CCND1)	2W9F	Regorafenib<br>Sorafenib<br>Nivolumab	-7.7<br>-7.5<br>-7.0	2.26893<br>3.17997<br>7.39482
3.	Fibronectin	1FNF	Regorafenib<br>Sorafenib<br>Nivolumab	-7.7<br>-7.5<br>-6.5	2.26893<br>3.17997<br>17.1962
4.	Peroxiredoxin 2 (PRDX2)	1QMV	Regorafenib<br>Sorafenib<br>Nivolumab	-7.5<br>-7.2<br>-6.5	3.17997<br>5.27625<br>17.1962
5.	Transgelin	1WYM	Sorafenib<br>Regorafenib<br>Nivolumab	-7.7<br>-7.6<br>-7.2	2.26893<br>2.6861<br>5.27625
6.	Protein disulfide-isomerase A3 (PDIA3)	2DMM<br><br><br>2H8L	Regorafenib<br>Sorafenib<br>Nivolumab<br>Regorafenib<br>Sorafenib<br>Nivolumab	-7.7<br>-7.5<br>-6.2<br>-9.2<br>-8.9<br>-6.8	2.26893<br>3.17997<br>28.5322<br>0.180428<br>0.29937<br>10.3641
7.	Insulin-like growth factor 2 (IGF2)	2H7T	Regorafenib<br>Sorafenib<br>Nivolumab	-8.1<br>-7.7<br>-7.3	1.15509<br>2.26893<br>4.45681
8.	S100A14	2M0R	Regorafenib<br>Sorafenib<br>Nivolumab	-8.0<br>-7.9<br>-7.0	1.36747<br>1.61889<br>7.39482
9.	Cyclin-dependent kinase 4 (CDK4)	2W99	Regorafenib<br>Sorafenib<br>Nivolumab	-8.9<br>-8.8<br>-7.0	0.29937<br>0.354412<br>7.39482
10.	Spondin-2	3D34	Regorafenib<br>Sorafenib<br>Nivolumab	-7.6<br>-7.5<br>-6.4	2.6861<br>3.17997<br>20.3579
11.	Bone marrow stromal antigen 2 (BST2)	3NWH	Sorafenib<br>Regorafenib<br>Nivolumab	-6.7<br>-6.2<br>-4.9	12.2696<br>28.5322<br>256.006
12.	C-reactive protein (CRP)	3PVN	Regorafenib<br>Sorafenib<br>Nivolumab	-9.1<br>-8.7<br>-5.9	0.213602<br>0.419575<br>47.3412
13.	Peroxiredoxin 6	5B6M	Sorafenib<br>Regorafenib	-7.5<br>-7.4	3.17997<br>3.76464

			Nivolumab	-6.3	24.101
14.	Prohibitin	6IQE	Regorafenib<br>Sorafenib<br>Nivolumab	-6.4<br>-6.4<br>-6.0	20.3579<br>20.3579<br>39.9888
15.	C-X-C motif chemokine ligand 10 (CXCL10)	1O7Y	Regorafenib<br>Sorafenib<br>Nivolumab	-6.4<br>-6.4<br>-6.0	20.3579<br>20.3579<br>39.9888
16.	Ezrin	1NI2	Sorafenib<br>Regorafenib<br>Nivolumab	-8.6<br>-8.4<br>-7.3	0.496719<br>0.696166<br>4.45681
17.	Retinoblastom a protein (RB1)	1AD6	Regorafenib<br>Sorafenib<br>Nivolumab	-8.3<br>-8.1<br>-6.9	0.824165<br>1.15509<br>8.75444
18.	Axis inhibition protein 1 (AXIN1)	1DK8	Regorafenib<br>Sorafenib<br>Nivolumab	-7.4<br>-7.4<br>-6.1	3.76464<br>3.76464<br>33.7782
19.	Insulin like growth factor 2 receptor (IGF2R)	1GP0	Regorafenib<br>Sorafenib<br>Nivolumab	-8.0<br>-7.9<br>-5.6	1.36747<br>1.61889<br>78.5493
20.	SMAD Family Member 2 (SMAD2)	1KHX	Sorafenib<br>Regorafenib<br>Nivolumab	-8.5<br>-8.1<br>-6.8	0.588047<br>1.15509<br>10.3641
21.	Aminoacylase 1	1Q7L	Sorafenib<br>Regorafenib<br>Nivolumab	-8.6<br>-8.4<br>-7.1	0.496719<br>0.696166<br>6.24635
22.	Tumor protein p53 (TP53)	1TUP	Regorafenib<br>Sorafenib<br>Nivolumab	-7.6<br>-7.6<br>-6.7	2.6861<br>2.6861<br>12.2696
23.	Activating transcription factor 4 (ATF-4)	1CI6	Sorafenib<br>Regorafenib<br>Nivolumab	-6.2<br>-6.1<br>-4.9	28.5322<br>33.7782<br>256.006
24.	Death receptor 5 (DR5)	1D0G	Regorafenib<br>Sorafenib<br>Nivolumab	-7.1<br>-6.9<br>-6.1	6.24635<br>8.75444<br>33.7782
25.	P27Kip1	1JSU	Sorafenib<br>Regorafenib<br>Nivolumab	-7.6<br>-7.5<br>-6.5	2.6861<br>3.17997<br>17.1962
26.	Eukaryotic Initiation Factor 2α (eIF2α)	1KL9	Sorafenib<br>Regorafenib<br>Nivolumab	-7.8<br>-7.6<br>-6.6	1.91655<br>2.6861<br>14.5255
27.	P58	2Y4T	Sorafenib<br>Regorafenib<br>Nivolumab	-7.8<br>-7.2<br>-6.6	1.91655<br>5.27625<br>14.5255
28.	Caspase 3	3KJF	Regorafenib<br>Sorafenib	-7.4<br>-7.1	3.76464<br>6.24635

			Nivolumab	-5.8	56.0454
29.	Growth arrest and DNA damage-inducible protein 34 (GADD34)	4XPN	Regorafenib<br>Sorafenib<br>Nivolumab	-5.3<br>-5.1<br>-4.9	130.33<br>182.662<br>256.006
30.	Glucose regulated protein 78 (GRP78)	5E85<br><br><br>5EVZ	Regorafenib<br>Sorafenib<br>Nivolumab<br>Sorafenib<br>Regorafenib<br>Nivolumab	-9.2<br>-8.8<br>-7.3<br>-9.5<br>-8.7<br>-7.4	0.180428<br>0.354412<br>4.45681<br>0.108743<br>0.419575<br>3.76464
31.	Calreticulin	5LK5	Regorafenib<br>Sorafenib<br>Nivolumab	-7.5<br>-7.5<br>-6.4	3.17997<br>3.17997<br>20.3579
32.	Protein disulfide isomerase (PDI)	6I7S	Regorafenib<br>Sorafenib<br>Nivolumab	-8.5<br>-8.1<br>-7.1	0.588047<br>1.15509<br>6.24635

Table S4: Primer sequences for qRT-PCR

Gene (Human)	Forward primer	Reverse primer
Ezrin	5’AGAAAGAGCAGATGATGCGCGAGA-3’	5’-AGGAGGGCAATCTTGGCAGTGTAT-3’
ACY-1	5’-GGCTGCATGAGGCTGTGTT-3’	5’-CTTGGCACTGGTTGGGATG-3
Rb1	5’-TTGGATCACAGCGATACAAACTT-3’	5’-AGCGCACGCCAATAAAGACAT-3’
Calreticulin	5’-TGGCGTGCTGGGCCTGGACCTCTGG-3’	5’-CCTCTTTGCGTTTCTTGTCTTCTTC-3’
β-Actin	5’-TGGCACCCAGCACAATGAA-3’	5’-CTAAGTCATAGTCCGCCTAGAAGCA-3’

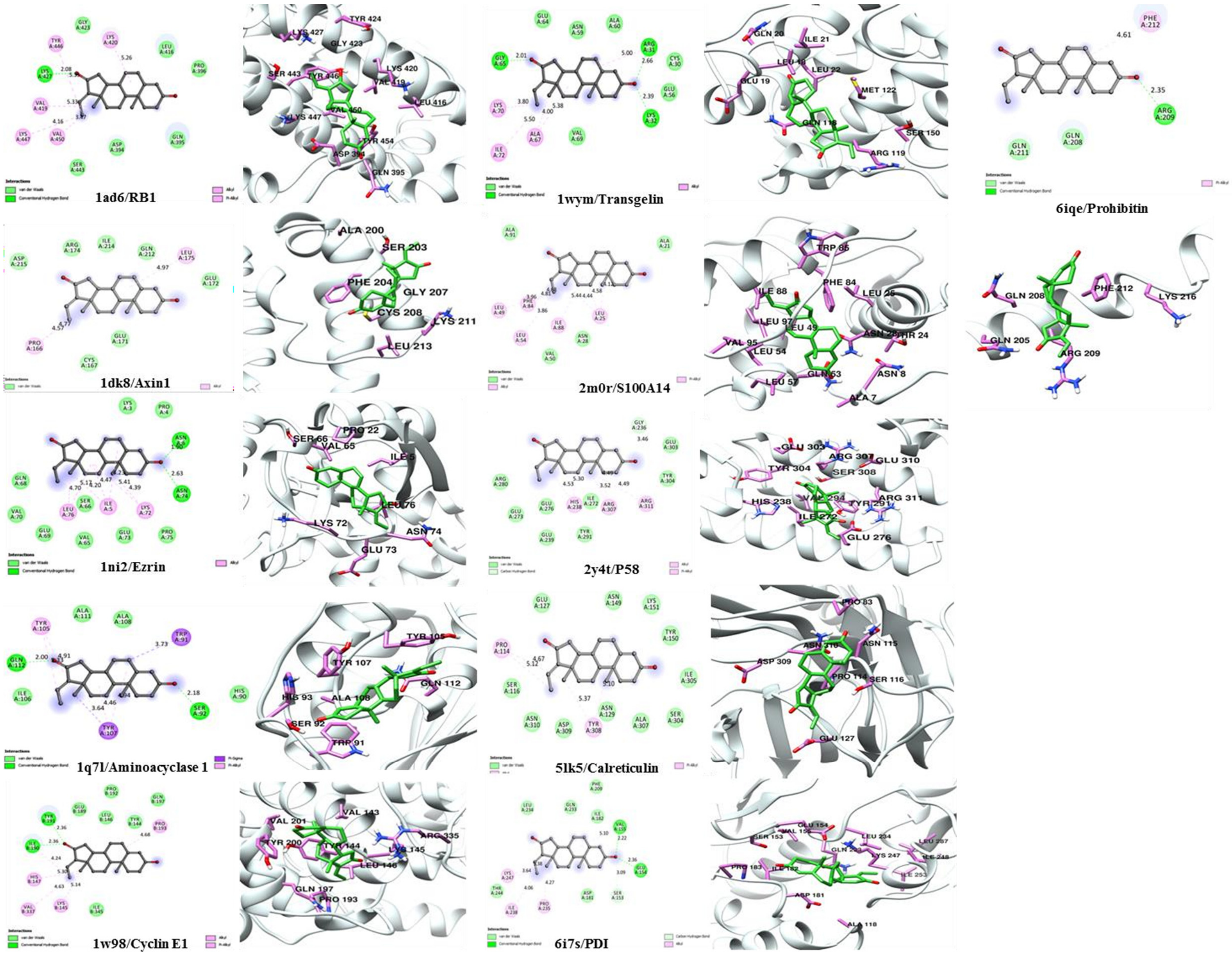

Figure S1: 2D interaction diagram (left) and 3D molecular dynamics simulation poses (right) for *z*-guggulsterone complexed with selected proteins: Cyclin E1, Ezrin, RB1, Aminoacylase, Transgelin, S100A14, Prohibitin, AXIN1, PDI, CRT and P58.

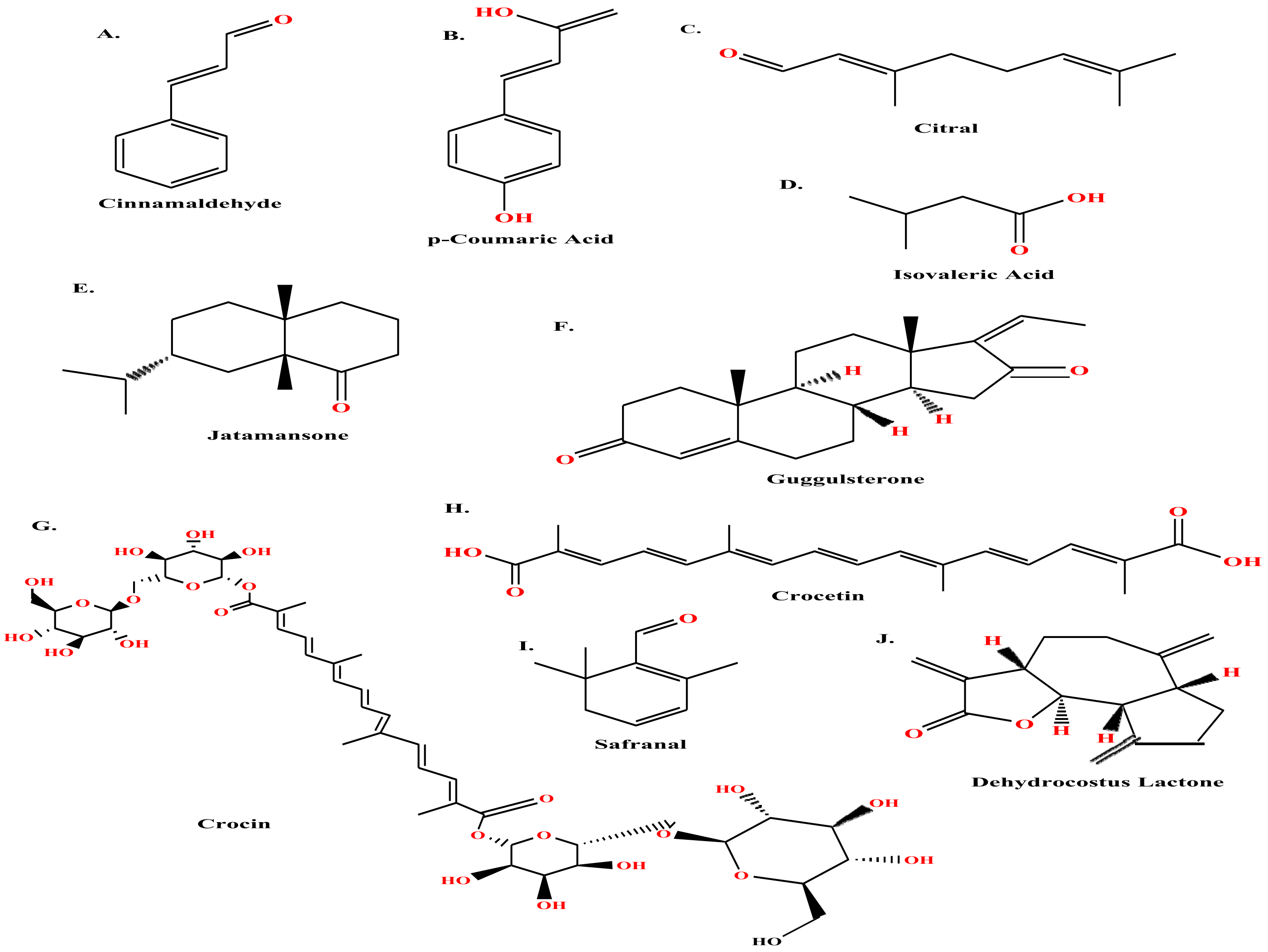

Figure S2: Chemical structure of the bioactive components of Duk.
